# Prognostic impact of pre-transplant chromosomal aberrations in peripheral blood of patients undergoing unrelated donor hematopoietic cell transplant for acute myeloid leukemia

**DOI:** 10.1038/s41598-021-94539-0

**Published:** 2021-07-22

**Authors:** Youjin Wang, Weiyin Zhou, Lisa J. McReynolds, Hormuzd A. Katki, Elizabeth A. Griffiths, Swapna Thota, Mitchell J. Machiela, Meredith Yeager, Philip McCarthy, Marcelo Pasquini, Junke Wang, Ezgi Karaesmen, Abbas Rizvi, Leah Preus, Hancong Tang, Yiwen Wang, Loreall Pooler, Xin Sheng, Christopher A. Haiman, David Van Den Berg, Stephen R. Spellman, Tao Wang, Michelle Kuxhausen, Stephen J. Chanock, Stephanie J. Lee, Theresa E. Hahn, Lara E. Sucheston-Campbell, Shahinaz M. Gadalla

**Affiliations:** 1 Clinical Genetics Branch, Division of Cancer Epidemiology and Genetics, National Cancer Institute, 9609 Medical Center Dr., Rockville, MD 20850 USA; 2 Cancer Genomics Research Laboratory, Frederick National Laboratory for Cancer Research, Leidos Biomedical Research, Inc, Frederick, MD USA; 3 Department of Medicine, Roswell Park Comprehensive Cancer Center, Buffalo, NY USA; 4 Department of Medicine, Medical College of Wisconsin, Milwaukee, WI USA; 5 College of Pharmacy, The Ohio State University, Columbus, OH USA; 6 Department of Preventive Medicine, University of Southern California, Los Angeles, CA USA; 7 Center for International Blood and Marrow Transplant Research, Minneapolis, MN USA; 8 Center for International Blood and Marrow Transplant Research, Medical College of Wisconsin, Milwaukee, WI USA; 9 Division of Biostatistics, Medical College of Wisconsin, Milwaukee, WI USA; 10 Clinical Research Division, Fred Hutchinson Cancer Research Center, Seattle, WA USA

**Keywords:** Prognostic markers, Epidemiology, Leukaemia, Cancer, Cancer stem cells, Cancer therapy

## Abstract

To improve risk stratification and treatment decisions for patients with acute myeloid leukemia (AML) undergoing hematopoietic cell transplantation (HCT). We used SNP-array data from the DISCOVeRY-BMT study to detect chromosomal aberrations in pre-HCT peripheral blood (collected 2–4 weeks before the administration of conditioning regimen) from 1974 AML patients who received HCT between 2000 and 2011. All aberrations detected in ≥ 10 patients were tested for their association with overall survival (OS), separately by remission status, using the Kaplan–Meier estimator. Cox regression models were used for multivariable analyses. Follow-up was through January 2019. We identified 701 unique chromosomal aberrations in 285 patients (7% of 1438 in complete remission (CR) and 36% of 536 not in CR). Copy-neutral loss-of-heterozygosity (CNLOH) in chr17p in CR patients (3-year OS = 20% vs. 50%, with and without chr17p CNLOH, p = 0.0002), and chr13q in patients not in CR (3-year OS = 4% vs. 26%, with and without chr13q CNLOH, p < 0.0001) are risk factors for poor survival. Models adjusted for clinical factors showed approximately three-fold excess risk of post-HCT mortality with chr17p CNLOH in CR patients (hazard ratio, HR = 3.39, 95% confidence interval CI 1.74–6.60, p = 0.0003), or chr13q CNLOH in patients not in CR (HR = 2.68, 95% CI 1.75–4.09, p < 0.0001). The observed mortality was mostly driven by post-HCT relapse (HR = 2.47, 95% CI 1.01–6.02, p = 0.047 for chr17p CNLOH in CR patients, and HR = 2.58, 95% CI 1.63–4.08, p < 0.0001 for chr13q CNLOH in patients not in CR. Pre-transplant CNLOH in chr13q or chr17p predicts risk of poor outcomes after unrelated donor HCT in AML patients. A large prospective study is warranted to validate the results and evaluate novel strategies to improve survival in those patients.

## Introduction

Acute myeloid leukemia (AML) is an aggressive disease with a variable response to therapy. Cytogenetics and more recently molecular profiling are the main predictors for disease prognosis and are used to guide patient therapeutic strategies^1–3^.

Allogeneic hematopoietic cell transplantation (HCT) is a potentially curative treatment option for AML, with the best outcomes observed in patients receiving HCT while in first complete remission (CR)^4^. The 3-year survival probabilities after HCT are 53%, 50%, and 27% for patients undergoing an unrelated donor HCT in first CR, in second or subsequent CR and those not in CR, respectively.^4^.

Recent studies have shown that the presence of pre-transplant measurable residual disease (MRD) is associated with increased risk of relapse and death after HCT in patients with AML^5–8^. Given the highly heterogenous nature of residual leukemia after induction therapy, it is of clinical interest to monitor genomic profiles in pre-transplant marrow or blood to evaluate aberrations that may further improve risk stratification for HCT. The use of highly sensitive methods such as SNP-array has been explored for chromosomal assessments in AML^9–11^. However, its possible clinical application in HCT is yet to be explored.

In this study, we used a high-resolution genome-wide SNP-array to characterize large clonal chromosomal aberrations in pre-HCT blood samples from a large cohort of AML patients undergoing unrelated donor HCT. We then evaluated whether detected chromosomal aberrations can aid prognostic classification of overall survival and risk of relapse after allogeneic transplant for AML.

## Results

### Patient characteristics

The median age at transplant was 48.2 (range 0.6–78.0) years. Fifty-three percent were males, 95% were Caucasians, 7.4% had therapy-related disease, and 73% were in complete remission. Chromosomal aberrations in pre-transplant blood samples were detected in 285 patients (14.4%). Patients with chromosomal aberrations were older at transplant (median = 54.0 vs. 47.2 years, p < 0.001) and had a poor KPS score (p < 0.001). As expected, clonal chromosomal aberrations were more frequently found in patients who received HCT while not in CR compared with those in CR (35.6% vs. 6.5%, p < 0.001). Participant clinical and transplant-related characteristics comparing those with and without any chromosomal aberrations are summarized in Table 1.

**Table 1:** Patient characteristics.

Characteristics	Chromosomal aberration, N (%)		P^1^
	Present (N = 285)	Absent (N = 1689)	
**Clinical factors**			
Recipient age			< 0.001
≤ 10	12 (4%)	60 (4%)	
10 < − 18	3 (1%)	93 (6%)	
18 < − 40	35 (12%)	452 (27%)	
40 < − 60	151 (53%)	791 (47%)	
> 60	84 (29%)	293 (17%)	
Recipient sex			0.09
Female	121 (42%)	809 (48%)	
Male	164 (58%)	880 (52%)	
Recipient race			0.54^2^
Caucasian	271 (95%)	1595 (94%)	
African–American	2 (1%)	33 (2%)	
Asian	4 (1%)	22 (1%)	
Other	3 (1%)	11 (1%)	
Missing/unknown	5 (2%)	28 (2%)	
Karnofsky Performance Score			< 0.001
90–100	147 (52%)	1053 (62%)	
< 90	110 (39%)	460 (27%)	
Missing	28 (10%)	176 (10%)	
Disease status prior to transplant			< 0.001
Not in complete remission	191 (67%)	345 (20%)	
In complete remission	94 (33%)	1344 (80%)	
Cytogenetics at diagnosis			0.007
Normal	58 (20%)	514 (30%)	
Abnormal	151 (53%)	782 (46%)	
No evaluation (metaphase)	2 (1%)	9 (1%)	
Unknown/missing	74 (26%)	384 (23%)	
**Transplant-related factors**			
Donor age			0.67
18–< 33	139 (49%)	871 (52%)	
33–< 50	129 (45%)	719 (43%)	
50–	17 (6%)	99 (6%)	
Donor sex			0.55
Female	96 (34%)	539 (32%)	
Male	189 (66%)	1150 (68%)	
Donor race			0.70
Caucasian	253 (89%)	1500 (89%)	
African–American	2 (1%)	30 (2%)	
Asian	5 (2%)	24 (1%)	
Other	19 (7%)	106 (6%)	
Missing/unknown	6 (2%)	29 (2%)	
In vivo T-Cell Depletion			0.83
ATG and/or Campath	87 (31%)	491 (29%)	
No ATG or Campath	184 (65%)	1121 (66%)	
Missing	14 (5%)	77 (5%)	
Donor–recipient CMV serostatus matching			0.14
Both negative	68 (24%)	452 (27%)	
Negative/positive	114 (40%)	623 (37%)	
Both positive	65 (23%)	335 (20%)	
Positive/negative	26 (9%)	227 (13%)	
Missing	12 (4%)	52 (3%)	
Graft type			0.50
Bone marrow	84 (29%)	532 (31%)	
Peripheral blood	201 (71%)	1157 (69%)	
GvHD prophylaxis			0.60
Tacrolimus + MMF ± others	54 (19%)	272 (16%)	
Other Tacrolimus based	149 (52%)	923 (55%)	
CSA based	75 (26%)	440 (26%)	
Other GVHD or no prophylaxis	7 (2%)	54 (3%)	
HLA matching, n (%)			0.50
10/10	260 (91%)	1519 (90%)	
< 10/10	25 (9%)	170 (10%)	
TBI in conditioning regimen			0.008
Yes	88 (31%)	660 (39%)	
No	197 (69%)	1029 (61%)	
Conditioning regimen intensity			0.16
Myeloablative	190 (67%)	1196 (71%)	
Reduced intensity	95 (33%)	493 (29%)	
Year of transplant			0.19
2000–2002	36 (13%)	173 (10%)	
2003–2005	93 (33%)	477 (28%)	
2006–2008	101 (35%)	673 (40%)	
2009–2011	55 (19%)	366 (22%)	

^1^Pearson Chi-square test unless otherwise specified.

^2^Fisher’s exact test.

### Detected clonal chromosomal aberrations by remission status at HCT

**Figure 1:**
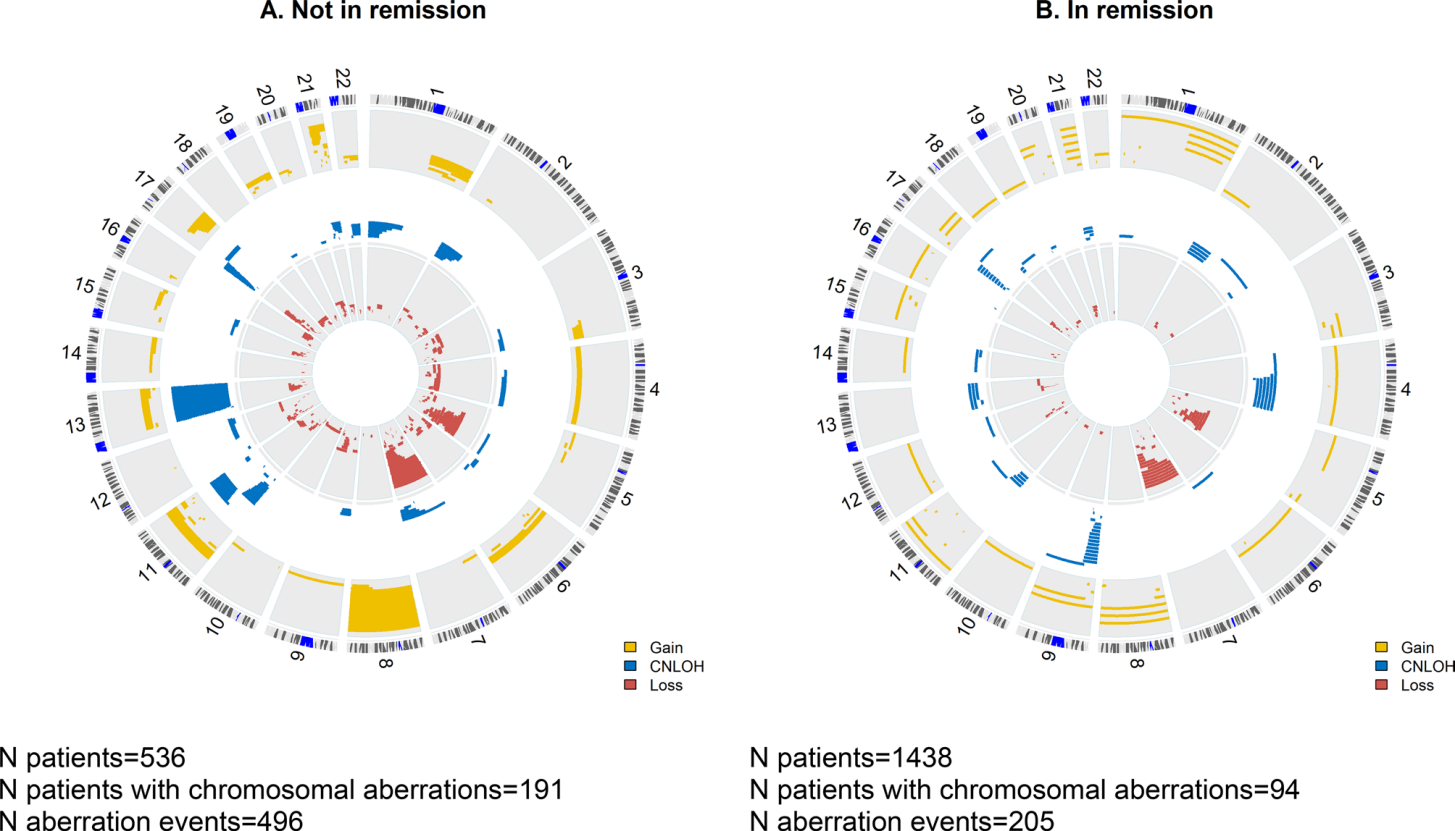
Genomic location and type of chromosomal aberrations among patients with AML. (**A**) Advanced disease (not in remission), (**B**) in complete remission; Yellow: copy-gain, blue: copy-neutral loss of heterozygosity (CNLOH), red: copy-loss. R package “OmicCircos” version 1.28.0^31^ was used to create the figures.

Figure 1A,B shows the type and location of detected chromosomal aberrations by remission status at transplant. In patients with advanced disease, a total of 496 aberrations were detected in 191 patients; among those, 49.7% had only one aberration, 33.5% had 2–4 aberrations and 16.8% had ≥ 5 aberrations. The median proportion of affected cells in patients with aberrations was 49% (interquartile range, IQR 29–79%). In patients undergoing HCT while in CR, 205 aberrations were detected in 94 (6.5%) patients; of them, 61.7% had one event, 30.9% had 2–4 events and 7.4% had ≥ 5 events. The median fraction of affected cells in patients with aberrations was 45% (IQR 28–62%). The frequency of patients with commonly detected aberrations is presented in Supplemental Table 1.

### Pre-transplant chromosomal aberrations associated with overall survival

**Figure 2:**
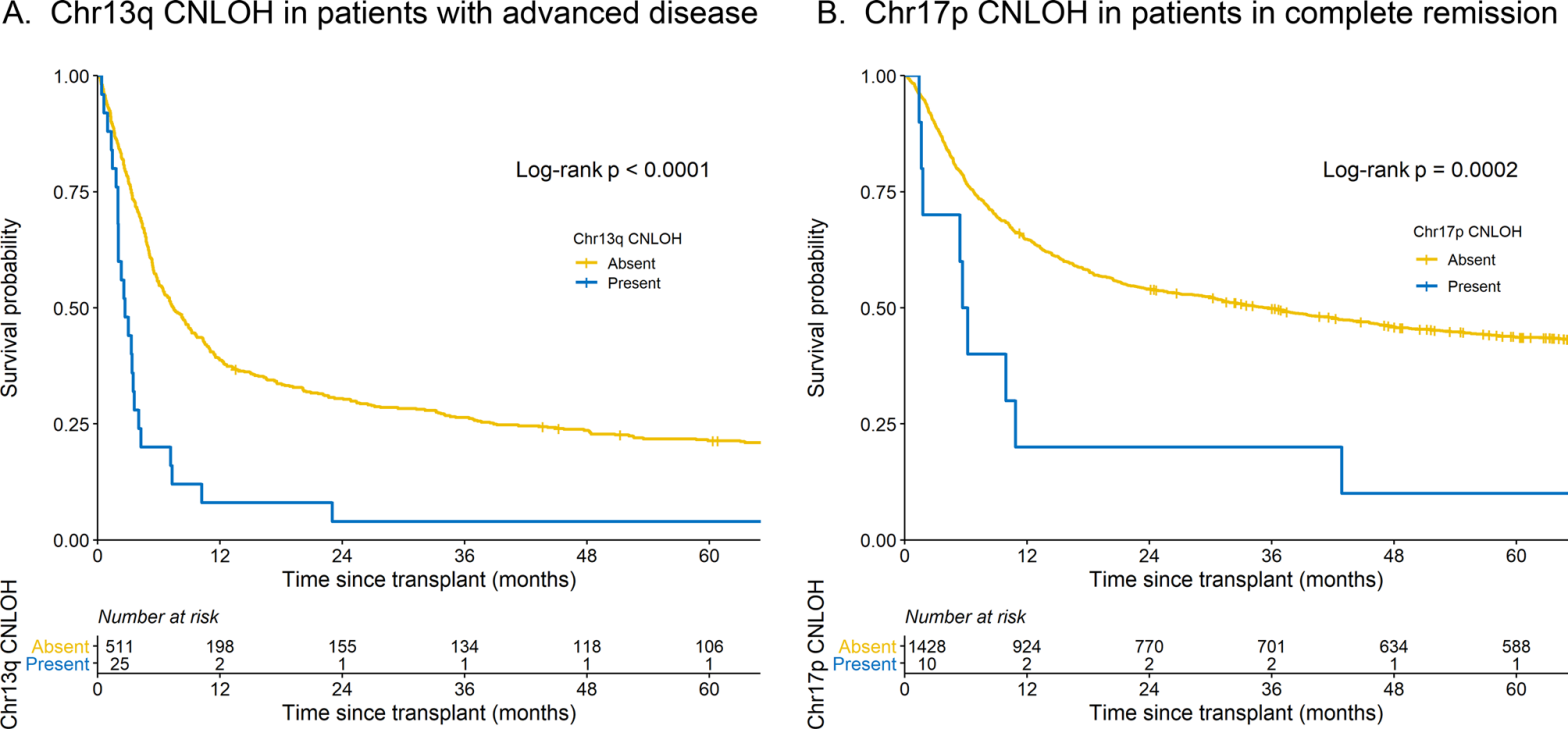
Survival probabilities after hematopoietic cell transplant in patients with AML: (**A**) Advanced disease (not in remission) by the presence or absence of chr13q CNLOH; (**B**) in complete remission by the presence or absence of chr17p CNLOH. Yellow: absent, blue: present.

Univariate analyses in patients with advanced disease showed poor post-HCT overall survival with the following aberrations: copy losses in chr5q (N = 24, log-rank p = 0.046), 17p (N = 16, p = 0.01), 12p (N = 12, p = 0.04), 15q (N = 9, p = 0.02), and CNLOH in 13q (N = 25, p < 0.0001). Only CNLOH chr13q remained statistically significantly associated with patient survival in a multivariable model with other aberrations and important clinical factors (Supplemental Table 2). The 3-year overall survival (OS) probabilities in patients with advanced disease with and without chr13q CNLOH were 4% vs. 26%, log-rank p < 0.0001, with a median survival of 2.7 vs. 7.4 months, and 96% vs. 73% of the death happened in the first year after HCT, respectively (Fig. 2A and Supplemental Table 3). Multivariable analysis adjusted for clinical factors, showed that patients with advanced disease and chr13q CNLOH were at three-fold higher risk of post-HCT mortality compared to those with advanced disease but not with chr13q CNLOH (HR = 2.68, 95% CI 1.75–4.09, p < 0.0001) (Table 2).

**Table 2:** Association between copy-neutral loss of heterozygosity in chr13q or chr17p and post-HCT outcomes in patients with AML.

Remission status	Copy neutral loss-of heterozygosity	All-cause mortality				Relapse			
		N event/total				N event/total			
		Present	Absent	HR (95% CI)	p	Present	Absent	HR (95% CI)	p
Not in CR	chr13q	24/25	429/511	2.68 (1.75–4.09)^a^	< 0.0001	20/25	313/510	2.58 (1.63–4.08)^b^	< 0.0001
In CR	chr17p	10/10	888/1428	3.39 (1.74–6.60)^c^	0.0003	5/10	505/1427	2.47 (1.01–6.02)^d^	0.047

^a^Model was adjusted for recipient race, Karnofsky Performance Status scores, study cohort, GvHD prophylaxis, donor–recipient CMV serostatus matching, and graft type.

^b^Model was adjusted for Karnofsky Performance Status scores, and graft type.

^c^Model was adjusted for recipient age, donor age, GvHD prophylaxis, Karnofsky Performance Status scores, donor-recipient CMV serostatus matching, and stratified on recipient sex, conditioning intensity, graft type, and year of transplant.

^d^Model was adjusted for conditioning intensity, donor sex, study cohort, and stratified on Karnofsky Performance Status scores.

In CR patients, the presence of CNLOHs in chromosomes 17p (N = 10, log-rank p = 0.0002), 9p (N = 13, p = 0.004), 2p (N = 4, p = 0.02), 11p (N = 4, p = 0.02), and copy losses in 5q (N = 10, p = 0.006), 13q (N = 6, p = 0.007) and 20q (N = 4, p = 0.04) were associated with poor post-HCT survival in univariate analyses. Among those, only CNLOH in chr17p was associated with a statistically significant excess risk of mortality in a model with other aberrations and clinical factors (Supplemental Table 2). The 3-year OS probabilities were 20% and 50%, in CR patients with and without chr17p CNLOH, respectively, log-rank p = 0.0002, median survival = 5.9 vs. 33.4 months, and 80% vs. 57% of the death happened in the first year after HCT, respectively (Fig. 2B and Supplemental Table 3). Multivariable analysis of associated mortality risk in CR patients with a chr17p CNLOH compared with those free of this aberration showed a HR = 3.39 (95% CI 1.74–6.60, p = 0.0003) (Table 2).

### Chromosomal aberration profile in patients with CNLOHs in chr13q or chr17p

**Figure 3:**
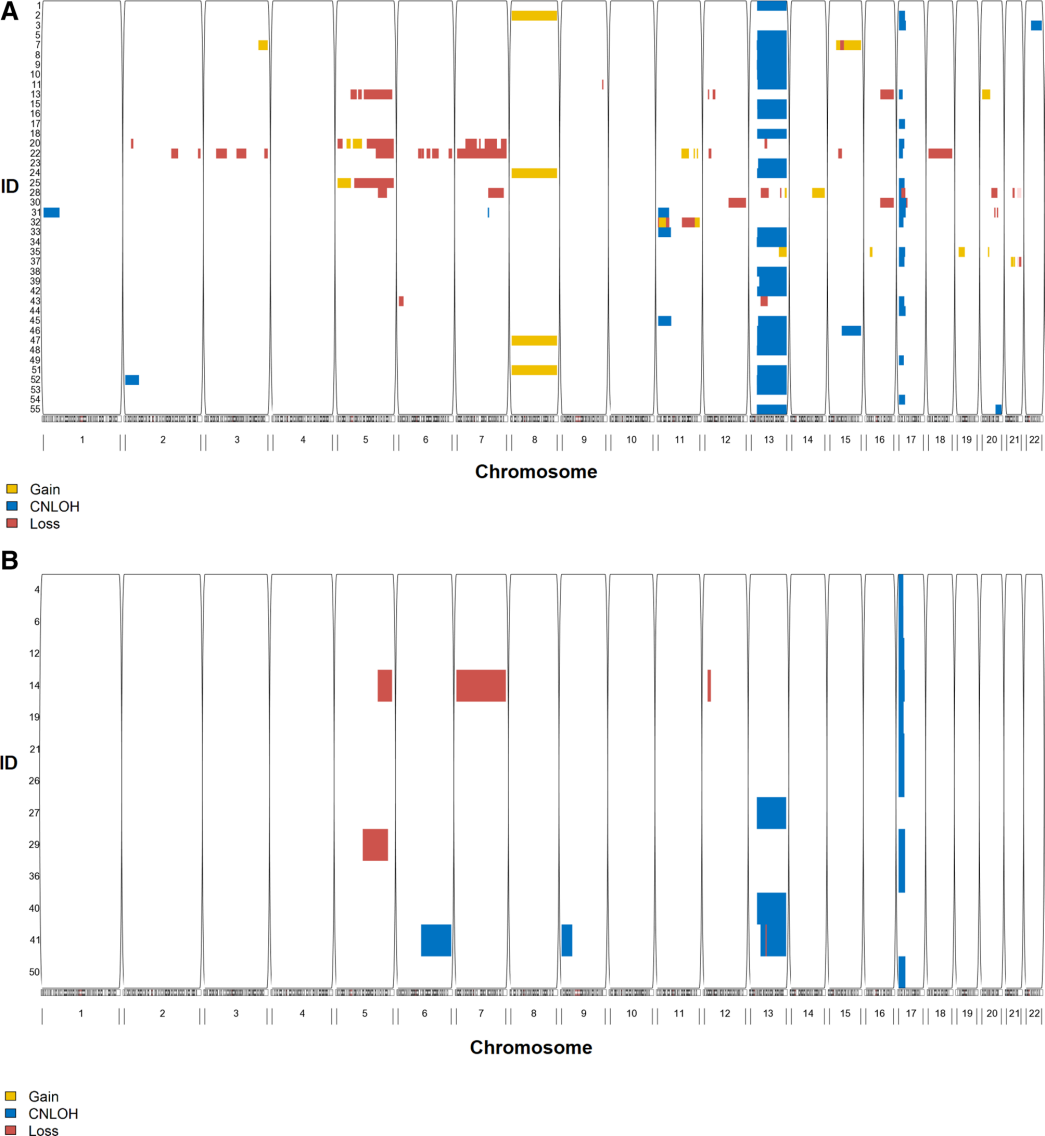
Aberration profile of AML patients with copy-neutral loss of heterozygosity in chr13q or chr17p. (**A**) Advanced disease (not in remission), (**B**) in complete remission; Yellow: copy-gain, blue: CNLOH, red: copy-loss. Partek Genomics Suite software version 7.0^30^ was used to create the figures.

A total of 28 patients had chr13q CNLOH, with the majority having advanced disease (N = 25). On the other hand, chr17p CNLOH was detected in 17 patients with advanced disease and 10 patients in CR. Figure 3A,B show aberration profile of patients with CNLOH in chr13q or chr17p by CR status. None of the patients had both aberrations regardless of their remission status. Thirty-nine percent (N = 11) of the patients with chr13q CNLOH carried aberrations in other chromosomes vs. 56% (N = 15) of those with chr17q CNLOH. Chromosomal regions affected by chr13q or 17p CNLOH in this study include *FLT3* in 13q12.2 and *TP53* in 17p13.1 (Supplemental Fig. 1A,B).

In 43 patients with chr13q or 17p CNLOH at HCT and cytogenetic information at AML diagnosis (N = 22 with 17p CNLOH and N = 21 with 13q CNLOH), 6/8 patients in CR with chr 17p CNLOH had complex karyotypes or deletion in chromosome 5 or 7. Yet, only one patient with chr13q CNLOH had either (Supplemental Fig. 2).

### Risk of relapse/disease progression in patients carrying either CNLOH in chr13q or chr17p

The 1- and 3-year cumulative incidences of relapse/disease progression in AML patients by remission status and chromosomal aberrations are summarized in Supplemental Table 3. In a multivariable model, the presence of CNLOHs in chr13q in patients with advanced disease or in chr17p in CR patients was associated with more than twofold increased risk of leukemia progression or relapse, respectively (HR = 2.58, 95% CI 1.63–4.08, p < 0.0001; and HR = 2.47, 95% CI 1.01–6.02, p = 0.047) (Table 2).

### CNLOH in chr13q or chr17p in patients with normal cytogenetics at AML diagnosis and their association with overall survival after HCT

In analysis restricted to patients with normal cytogenetics at AML diagnosis (N = 572), approximately one-quarter (N = 157, 27.4%) received HCT while not in CR, with CNLOHs in chr13q (N = 13, 8.3%) the most frequently detected aberration in this group. Among CR patients with normal cytogenetics at diagnosis, 5% had chromosomal aberrations at HCT but none had chr17p CNLOH. Supplemental Fig. 3A,B summarize the chromosomal aberration profile in patients with normal cytogenetics at AML diagnosis by CR status. Similar to results from the full cohort, the presence of chr13q CNLOH in patients with advanced disease in this subgroup analysis was associated with excess risk of post-HCT mortality (HR = 2.78, 95% CI 1.48–5.21, p = 0.002) (Supplemental Table 4).

### CNLOH in chr13q or chr17p by patient age and their association with overall survival after HCT

Supplemental Fig. 4A–F show chromosomal aberration profile by patient age group. Notably, only one pediatric patient (≤ 18 years) had chr13q CNLOH and none carried aberrations in chr17. CNLOH in chr17p in CR patients was only observed in those older than 40 years. The effect of chr13q CNLOH on OS in patients with advanced disease was consistent across age groups (HR = 2.31, 95% CI 1.02–5.24, p = 0.045, and HR = 2.63, 95% CI 1.56–4.43, p = 0.0003 in those ≤, or > 40 years) (Supplemental Table 4). The absence of chr17p CNLOH in young CR patients did not allow for age-specific analysis. In analysis restricted to adult patients (> 18), chr13q CNLOH was associated with an approximately threefold increased risk of death and relapse in patients with advanced disease (OS: HR = 2.83, 95% CI 1.83–4.35, p < 0.0001; relapse: HR = 2.74, 95% CI 1.71–4.40, p < 0.0001). Chr17p CNLOH was associated with a threefold increase risk of mortality and more than a twofold increased relapse risk in CR patients (OS: HR = 3.25, 95% CI 1.65–6.37, p = 0.001; relapse: HR = 2.46, 95% CI 1.01–6.01, p = 0.048).

### CNLOH in chr13q or chr17p and their association with outcomes after HCT in patients with de novo AML

Supplemental Fig. 5A–D show chromosomal aberration profile in patients with de novo and treatment-related AML stratified by remission status. In de novo AML (N = 1611), the most frequent aberration in patients in advanced disease (N = 455) was chr13q CNLOH (N = 23, 5.1%), followed by chr5q loss (N = 18, 4%), and in those in CR (N = 1156) was CNLOH in chr9p (N = 11, 1%), followed by chr17p CNLOH (N = 7, 0.6%) and chr5q loss (N = 7, 0.6%). In treatment-related AML (N = 145), the most common aberration was chr5q loss in advanced disease (N = 4, 15.4%). In an analysis restricted to patients with de novo AML (N = 1611), we observed similar results to that of the full cohort. In patients with advanced disease, the presence of chr13q CNLOH was associated with approximately 3-folds increased risks of death or leukemia progression (OS: HR = 2.82, 95% CI 1.81–4.40, p < 0.0001; progression: HR = 2.86, 95% CI 1.78–4.62, p < 0.0001) (Supplemental Table 5). In CR patients, the presence of chr17p CNLOH was associated with threefold increased risk of death (HR = 3.14, 95% CI 1.38–7.15, p = 0.01) and twofold excess risk of relapse, although not statistically significant (HR = 2.33, p = 0.15). Small number of patients with treatment-related AML (N = 145) did not allow for robust outcome analyses.

## Discussion

In this large study of 1974 AML patients who underwent unrelated donor HCT, we showed that CNLOH in chr17p or chr13q (detected in approximately 3% of the patients) can identify new high-risk groups. The 3-year OS for CR patients carrying chr17p CNLOH was similar to those with advanced disease in the absence of chr13q CNLOH (3 years OS = 20% vs. 26%). Yet, harboring chr13q CNLOH in patients with advanced disease resulted in a detrimental outcome with a 3-year OS of 4% (with almost all death happened in the first year after HCT). The observed poor survival associated with such aberrations were primarily driven by the excess risk of relapse or disease progression. Although relatively small numbers, poor survival associated with CNLOH in chr17p or chr13q can refine pre-HCT risk stratification and guide precision medicine strategy in HCT decisions for patients with AML.

Responses to induction therapy dictated HCT prognosis in patients with AML for many years, with patients receiving HCT in first CR having the best prognosis and those with advanced disease with the worst outcome^4^. Recent studies showed a prognostic improvement when considering measurable residual disease in patients with morphological remission^12–14^. It is plausible that the observed inferior survival associated with CNLOH in chr17p or chr13q in our study is a reflection of residual leukemia or its aggressive phenotype. We showed that these aberrations affect genomic regions harboring *TP53* and *FLT3*, respectively, both known to be associated with risk of leukemia relapse and poor prognosis^15,16^. The presence of mutated *TP53* or high allelic ratio of *FLT3-ITD* at AML diagnosis are included in the adverse risk category of the European Leukemia Net 2017 (ELN-2017)^2^. We were unable to examine the role of ELN risk scheme in our analysis due to the lack of information but the presence of chr13q CNLOH in patients with *FLT3-ITD* was associated with a shorter survival (median OS = 2.3 vs.10.1 months, p = 0.009, in patients with and without chr13q CNLOH, respectively) in one study^17^. It is possible that the loss-of-heterozygosity in those genomic regions are subsequent events to mutations in *TP53* or *FLT3* that may modify patient outcome. If valid, pre-HCT testing for clonal CNLOH in chr17p or chr13q may guide the identification of patients at highest risk of inferior outcomes among patients harboring somatic mutations in *TP53* and *FLT3*, a hypothesis that warrants investigation.

In line with previous literature in AML^17^, our finding showed that chr13q CNLOH was detected primarily in patients with advanced disease (25/28); this was also true for the subset of patients who had normal cytogenetics at AML diagnosis. The observed detrimental post-HCT outcome in patients with advanced disease and chr13q CNLOH (median survival = 2.7 months; 3-year OS = 4%) need to be validated in a larger study as it questions the role of HCT in those patients and calls for careful risk–benefit assessment for this vulnerable patient population. Also, the presence of CNLOH in chr13q at HCT further stratified patients who were originally classified as intermediate risk based with normal cytogenetics at AML diagnosis. All those patients did not achieve CR and had an approximately three-fold risk of post-HCT leukemia relapse when compared to advanced disease patients free of such aberration. This observation calls for a longitudinal evaluation of chromosomal loss-of heterozygosity in AML patients with normal cytogenetics to understand their dynamics through the disease treatment course.

Surprisingly, the effect of chr17p CNLOH on post-HCT survival was restricted to CR patients. It is possible that the higher frequency of reduced intensity regimens in CR patients with chr17p CNLOH (60% in CR vs. 18% in advanced disease) contributed to this observation. Alternatively, the use of fludarabine-based regimen (in 80% of CR patients with chr17p CNLOH) was less effective. *In-vitro* studies suggested that cells with chr17 abnormalities are resistant to fludarabine-induced apoptosis^18,19^. A recent CIBMTR study in myelodysplastic syndrome showed that the presence of *TP53* mutation was associated with an approximately twofold increased risk in post-HCT mortality (HR = 1.71, p < 0.001)^20^; this was not modified by conditioning regimen intensity. A prospective study is needed to determine the best conditioning regimen strategy for those patients.

In the current study, almost none of the 168 pediatric AML patients carried either of our identified prognostic aberrations; small sample size may be a limiting factor. Yet, a recent large study of young AML patients (age at diagnosis ranging between days and 29 years) showed different molecular landscape than that of adult AML with distinct *FLT3* mutations but almost no *TP53* mutations^21^.

In the current study, we found no associations between pre-HCT SNP array-detected copy loss in chr5q or chr7q and patient post-HCT survival in either advanced or CR patients. Monosomy 7 and monosomy 5 or 5q deletion are known adverse cytogenetic prognostic markers for AML^2^. Of note, 26% of patients with chr17p CNLOH (N = 7, 2 in CR and 5 not in CR) had pre-HCT SNP-array detected copy loss in either chr5 or chr7, while no patient with 13q CNLOH had such aberrations. This was also consistent in the subset of patients with available cytogenetic information at AML diagnosis, where approximately 63% of the CR patients with chr17p CNLOH had deletions in chr5 or chr7 (N = 5/8), but only one patient with chr13q CNLOH did. Taken together, this suggests that the observed association with chr13q CNLOH is mostly independent of other known risk cytogenetic findings.

The clinical usefulness of SNP microarray platform in hematological malignancies is widely supported particularly as technology advances and cost drops^22^. Its possible use to guide HCT decision-making is still under investigation. Using the same technology, we previously showed a possible value to its use for transplant decisions in patients with Fanconi anemia^23^, an inherited marrow failure syndrome caused by defects in DNA repair genes, and acute lymphoblastic leukemia^24^.

The strengths of this study include the large sample size, the use of the genome-wide sensitive array, and the availability of well-captured clinical and outcome data. Yet, our study was limited by the unavailability of serial samples, which restrict our ability to explain whether those detected alterations are markers of residual disease or clonal drivers. Our analyses focused on aberrations observed in ≥ 10 patients, a larger study is warranted to validate our findings and evaluate less frequent aberrations. The study also lacked information on pre-HCT measurable residual disease, ELN risk classification, and prognostic mutations. SNP array analysis was completed using peripheral blood DNA; the use of bone marrow samples to validate our findings is warranted.

In conclusion, we showed poor survival associated with CNLOHs in chr13q and 17p in patients with AML. Further, larger scale study with diagnostic and pre-HCT samples for both genomic sequencing and SNP array analyses are warranted to elucidate the possible interplay between somatic point mutations and chromosomal alterations in AML pathogenesis, prognosis, and treatment response.

## Methods

### Study participants

This study included patients with AML (N = 1974) who were part of the DISCOVeRY-BMT (Determining the Influence of Susceptibility COnveying Variants Related to one-Year mortality after BMT) study (details are available elsewhere)^25^. AML patients in this study received unrelated donor HCT between 2000 and 2011. Blood samples and clinical data were available from the Center for International Blood and Marrow Transplant Research (CIBMTR) database and biorepository (https://www.cibmtr.org).

All patients provided informed consent for inclusion in the CIBMTR database and research repository. The study was exempted from full IRB review by Roswell Park Comprehensive Cancer Center Institutional Review Board and the NIH Office for Human Research Protections as all data for the analyses were de-identified.

### Detection of chromosomal aberrations

DNA was extracted from peripheral whole blood samples collected 2–4 weeks prior to HCT conditioning regimen administration. We used SNP-array genotype data generated by the HumanOmniExpress-12v1_A BeadChip to calculate the log_2_ R ratio (LRR) and B allele frequency (BAF). LRR is computed as the log_2_ ratio of observed to expected signal intensity for each SNP; LRR > 0 indicates copy number gain, whereas LRR < 0 indicates copy number loss^26^. BAF is calculated as the frequency of the B allele at a given biallelic SNP, where BAF = 0.5 indicates a heterozygous genotype. A deviation from heterozygosity in the BAF without LRR changes indicates a copy-neutral loss of heterozygosity (CNLOH)^27^. We used quantile normalization and GC and CpG waves correction to improve accuracy, as described previously^28^. We used a custom software pipeline that included BAF Segmentation software^29^. All potential events were plotted, and false positive calls were removed after manual review. We only included chromosomal aberrations ≥ 2 Mb to minimize false positive discovery^28^. The detection method we used have shown to yield higher sensitivity and specificity for detecting allelic imbalances when compared with other CNV detection methods^29^. Partek Genomics Suite software, version 7.0^30^ (Partek Inc., St. Louis, MO, USA, https://www.partek.com/partek-genomics-suite/) and R package “OmicCircos”, version 1.28.0^31^ (https://bioconductor.org/packages/OmicCircos/) were used for visual representation of chromosomal aberration data.

### Study end points

Two end points were tested for this study: (1) overall survival (OS) defined as time from date of HCT to death from any cause or last follow-up, and (2) Leukemia relapse or disease progression. Complete remission in this study is primarily based on hematologic remission.

### Statistical analysis

We compared differences in clinical and transplant-related factors between patients with and without aberrations using chi-square or Fisher’s exact test, as appropriate. All outcome analyses were completed separately by patient CR status to account for the differences in clinical management, HCT outcomes, and frequencies and types of detected aberrations between the two groups (N in CR = 1438 and N not in CR, i.e. advanced disease = 536). To identify chromosomal aberrations of prognostic effect, we first used the Kaplan–Meier estimator to calculate OS with each chromosomal aberration that occurred in ≥ 10 patients in the full cohort. All aberrations with statistically significant associations in the univariate models were then included in a multivariable Cox model to evaluate the independent effect of each controlling for other aberrations and important clinical factors. Aberrations that remained statistically significant from the previous step were tested for their associations with OS and relapse or disease progression in multivariable Cox proportional hazards models. Clinical factors included in the models were selected using a stepwise selection strategy with a threshold of p < 0.25 for entry and p < 0.15 for stay. Proportional hazard assumptions were examined using Schoenfeld residuals and violation was accounted for through stratification for non-proportional hazards effect. We used the cause-specific hazard analysis for relapse/disease progression with transplant related mortality treated as competing risk. Follow-up started at date of HCT and ended at outcome under-study, last follow-up, or in January 2019. The models for OS were adjusted for the following: (1) in CR patients: recipient and donor age, graft-versus-host disease (GvHD) prophylaxis, Karnofsky Performance Status (KPS) scores, donor–recipient cytomegalovirus (CMV) serostatus matching, and stratified on recipient sex, conditioning regimen intensity, graft type, and year of transplant, and (2) in patients with advanced disease: recipient race, KPS scores, study cohort, GvHD prophylaxis, donor–recipient CMV serostatus matching, and graft type. Relapse/disease progression models were adjusted for: (1) conditioning regimen intensity, donor sex, study cohort, and stratified on KPS scores for CR patients, and (2) KPS scores, and graft type in patients with advanced disease.

All statistical analyses were conducted using R version 4.0.2^32^ (R Foundation for Statistical Computing, Vienna, Austria, https://www.R-project.org) and SAS software version 9.4^33^ (SAS Institute, Inc., Cary, NC, USA, https://www.sas.com/en_us/home.html).

## Supplementary Information

Supplementary Information.
