# Supplementary material for: Prognostic impact of pre-transplant chromosomal aberrations in peripheral blood of patients undergoing unrelated donor hematopoietic cell transplant for acute myeloid leukemia
Source: Sci Rep. 2021 Jul 22;11:15004. doi: 10.1038/s41598-021-94539-0 (PMC8298542; doi:10.1038/s41598-021-94539-0)
Supplement: Supplementary file 1 — Supplementary Information. [file 41598_2021_94539_MOESM1_ESM.docx]

**Table of Contents**

[**Supplemental Table 1.** Chromosomal aberrations in pre-transplant blood samples detected in ≥ 10 patients with AML. 2](#_Toc74905654)

[**Supplemental Table 2.** Multivariable analysis of all-cause mortality after HCT in patients with AML including all chromosomal aberrations that showed significant associations in univariate analyses. 3](#_Toc74905655)

[**Supplemental Table 3.** Overall survival probabilities and cumulative incidence of post-HCT relapse by remission status and CNLOH in chr13q and chr17p at transplant in AML patients. 4](#_Toc74905656)

[**Supplemental Table 4.** Subgroup analysis of the association between CNLOH in chr13q and outcomes after HCT in patients with advanced disease at the time of transplant. 5](#_Toc74905657)

[**Supplemental Table 5.** Subgroup analysis of the association between CNLOH in chr13q or 17p and outcomes after HCT in patients with *De Novo* AML. 6](#_Toc74905658)

[**Supplemental Figure 1.** Chromosomal regions commonly affected by CNLOH in chr13q or 17p. 7](#_Toc74905659)

[**Supplemental Figure 2.** Cytogenetics at diagnosis reported by transplant centers in 43 patients with CNLOH in chromosome 13q or 17p 8](#_Toc74905660)

[**Supplemental Figure 3.** Genomic location and type of chromosomal aberrations among AML patients with normal cytogenetics at diagnosis 9](#_Toc74905661)

[**Supplemental Figure 4.** Genomic location and type of chromosomal aberrations among AML patients by age at transplant. 10](#_Toc74905662)

[**Supplemental Figure 5.** Genomic location and type of chromosomal aberrations among AML patients by AML type. 11](#_Toc74905663)

| **Supplemental Table 1.** Chromosomal aberrations in pre-transplant blood samples detected in ≥ 10 patients with AML. | | | | | | | | | | | | | | | | | | | | | | |
| --- | --- | --- | --- | --- | --- | --- | --- | --- | --- | --- | --- | --- | --- | --- | --- | --- | --- | --- | --- | --- | --- | --- |
| Chromosomal aberration | All  (N=1974) | | Disease status at HCT | | | | Cytogenetics at diagnosis | | | | | | | | Age at transplant | | | | | | | |
|  |  |  |  |  |  |  | Normal | | | | Abnormal | | | | 0-40 years | | | | >40 years | | | |
|  |  |  | Not in CR  (N=536) | | In CR  (N=1438) | | Not in CR  (N=157) | | In CR  (N=415) | | Not in CR  (N=238) | | In CR  (N=695) | | Not in CR  (N=158) | | In CR  (N=497) | | Not in CR  (N=378) | | In CR  (N=941) | |
|  | N | % | N | % | N | % | N | % | N | % | N | % | N | % | N | % | N | % | N | % | N | **%** |
| Chr5q LOSS | 34 | 1.7 | 24 | 4.5 | 10 | 0.7 | 1 | 0.6 | 0 | 0 | 20 | 8.4 | 9 | 1.3 | 3 | 1.9 | 2 | 0.4 | 21 | 5.6 | 8 | 0.9 |
| Chr13q CNLOH | 28 | 1.4 | 25 | 4.7 | 3 | 0.2 | 13 | 8.3 | 0 | 0 | 7 | 2.9 | 1 | 0.1 | 8 | 5.1 | 0 | 0 | 17 | 4.5 | 3 | 0.3 |
| Chr17p CNLOH | 27 | 1.4 | 17 | 3.2 | 10 | 0.7 | 1 | 0.6 | 0 | 0 | 13 | 5.5 | 8 | 1.2 | 1 | 0.6 | 0 | 0 | 16 | 4.2 | 10 | 1.1 |
| Chr7pq LOSS | 23 | 1.2 | 16 | 3.0 | 7 | 0.5 | 0 | 0 | 0 | 0 | 10 | 4.2 | 4 | 0.6 | 3 | 1.9 | 1 | 0.2 | 13 | 3.4 | 6 | 0.6 |
| Chr17p LOSS | 22 | 1.1 | 16 | 3.0 | 6 | 0.4 | 2 | 1.3 | 0 | 0 | 10 | 4.2 | 4 | 0.6 | 2 | 1.3 | 1 | 0.2 | 14 | 3.7 | 5 | 0.5 |
| Chr7q LOSS | 22 | 1.1 | 15 | 2.8 | 7 | 0.5 | 0 | 0 | 0 | 0 | 11 | 4.6 | 4 | 0.6 | 2 | 1.3 | 1 | 0.2 | 13 | 3.4 | 6 | 0.6 |
| Chr8pq GAIN | 20 | 1.0 | 17 | 3.2 | 3 | 0.2 | 1 | 0.6 | 0 | 0 | 13 | 5.5 | 3 | 0.4 | 7 | 4.4 | 0 | 0 | 10 | 2.6 | 3 | 0.3 |
| Chr12p LOSS | 18 | 0.9 | 12 | 2.2 | 6 | 0.4 | 0 | 0 | 1 | 0.2 | 9 | 3.8 | 5 | 0.7 | 1 | 0.6 | 0 | 0 | 11 | 2.9 | 6 | 0.6 |
| Chr21q GAIN | 17 | 0.9 | 11 | 2.1 | 6 | 0.4 | 0 | 0 | 1 | 0.2 | 9 | 3.8 | 5 | 0.7 | 1 | 0.6 | 3 | 0.6 | 10 | 2.6 | 3 | 0.3 |
| Chr9p CNLOH | 16 | 0.8 | 3 | 0.6 | 13 | 0.9 | 0 | 0 | 3 | 0.7 | 2 | 0.8 | 7 | 1 | 1 | 0.6 | 1 | 0.2 | 2 | 0.5 | 12 | 1.3 |
| Chr11p CNLOH | 15 | 0.8 | 11 | 2.1 | 4 | 0.3 | 7 | 4.5 | 0 | 0 | 2 | 0.8 | 1 | 0.1 | 3 | 1.9 | 1 | 0.2 | 8 | 2.1 | 3 | 0.3 |
| Chr13q LOSS | 15 | 0.8 | 9 | 1.7 | 6 | 0.4 | 1 | 0.6 | 1 | 0.2 | 5 | 2.1 | 3 | 0.4 | 1 | 0.6 | 0 | 0 | 8 | 2.1 | 6 | 0.6 |
| Chr20q LOSS | 12 | 0.6 | 8 | 1.5 | 4 | 0.3 | 0 | 0 | 0 | 0 | 6 | 2.5 | 4 | 0.6 | 0 | 0 | 1 | 0.2 | 8 | 2.1 | 3 | 0.3 |
| Chr2p CNLOH | 11 | 0.6 | 7 | 1.3 | 4 | 0.3 | 4 | 2.5 | 2 | 0.5 | 0 | 0 | 1 | 0.1 | 1 | 0.6 | 0 | 0 | 6 | 1.6 | 4 | 0.4 |
| Chr11q CNLOH | 11 | 0.6 | 10 | 1.9 | 1 | 0.1 | 1 | 0.6 | 0 | 0 | 8 | 3.4 | 1 | 0.1 | 2 | 1.3 | 0 | 0 | 8 | 2.1 | 1 | 0.1 |
| Chr9q LOSS | 10 | 0.5 | 9 | 1.7 | 1 | 0.1 | 1 | 0.6 | 1 | 0.2 | 8 | 3.4 | 0 | 0 | 1 | 0.6 | 1 | 0.2 | 8 | 2.1 | 0 | 0 |
| Chr15q LOSS | 10 | 0.5 | 9 | 1.7 | 1 | 0.1 | 1 | 0.6 | 0 | 0 | 5 | 2.1 | 1 | 0.1 | 2 | 1.3 | 0 | 0 | 7 | 1.9 | 1 | 0.1 |
| Chr1q GAIN | 10 | 0.5 | 7 | 1.3 | 3 | 0.2 | 1 | 0.6 | 0 | 0 | 4 | 1.7 | 2 | 0.3 | 1 | 0.6 | 0 | 0 | 6 | 1.6 | 3 | 0.3 |

| **Supplemental Table 2.** Multivariable analysis of all-cause mortality after HCT in patients with AML including all chromosomal aberrations that showed significant associations in univariate analyses. | | |
| --- | --- | --- |
| 1. Patients not in remission |  |  |
| Aberration | HR (95% CI)^1^ | P |
| Chr5q copy loss | 1.39 (0.86-2.24) | 0.18 |
| Chr12p copy loss | 1.69 (0.94-3.07) | 0.08 |
| Chr15q copy loss | 1.38 (0.63-3.05) | 0.42 |
| Chr17p copy loss | 1.65 (0.94-2.88) | 0.08 |
| Chr13q CNLOH | 2.77 (1.81-4.24) | <0.0001 |
| 1. Patient in remission |  |  |
| Aberration | HR (95% CI)^2^ | P |
| Chr5q copy loss | 1.35 (0.62-2.91) | 0.45 |
| Chr13q copy loss | 1.30 (0.47-3.58) | 0.62 |
| Chr20q copy loss | 1.41 (0.42-4.70) | 0.58 |
| Chr2p CNLOH | 1.93 (0.66-5.69) | 0.23 |
| Chr9p CNLOH | 1.34 (0.66-2.70) | 0.42 |
| Chr11p CNLOH | 2.36 (0.83-6.71) | 0.11 |
| Chr17p CNLOH | 3.22 (1.60-6.48) | 0.001 |
| 1. The model for post-transplant mortality was adjusted for recipient race, KPS scores, DISCOVeRY-BMT cohort, GvHD prophylaxis, donor-recipient CMV serostatus, and graft type. 2. The model for post-transplant mortality was adjusted for recipient age, donor age, GvHD prophylaxis, KPS scores, donor-recipient CMV serostatus and stratified on recipient sex, conditioning regimen, graft type and year of transplant. | | |

| **Supplemental Table 3.** Overall survival probabilities and cumulative incidence of post-HCT relapse by remission status and CNLOH in chr13q and chr17p at transplant in AML patients. | | | | | | |
| --- | --- | --- | --- | --- | --- | --- |
|  | Not in remission | | | In remission | | |
|  | with  13q CNLOH | without  13q CNLOH |  | with  17p CNLOH | without  17p CNLOH |  |
| Outcomes | P (95% CI) | P (95% CI) | p-value | P (95% CI) | P (95% CI) | p-value |
| Overall survival |  |  | <0.0001 |  |  | 0.0002 |
| N event/total | 24/25 | 429/511 |  | 10/10 | 888/1428 |  |
| 1-year | 8 (1-22)% | 39 (35-43)% |  | 20 (2-49)% | 65 (62-67)% |  |
| 3-year | 4 (0-15)% | 26 (23-30)% |  | 20 (2-49)% | 50 (47-52)% |  |
| 5-year | 4 (0-15)% | 21 (18-25)% |  | 10 (0-35)% | 44 (41-46)% |  |
| Relapse |  |  | 0.03 |  |  | 0.29 |
| N event/total | 20/25 | 313/510 |  | 5/10 | 505/1427 |  |
| 1-year | 80 (56-92)% | 56 (51-60)% |  | 40 (10-69)% | 26 (24-29)% |  |
| 3-year | 80 (56-92)% | 60 (55-64)% |  | 50 (14-78)% | 32 (30-34)% |  |

| **Supplemental Table 4.** Subgroup analysis of the association between CNLOH in chr13q and outcomes after HCT in patients with advanced disease at the time of transplant. | | | | | | | | | |
| --- | --- | --- | --- | --- | --- | --- | --- | --- | --- |
|  |  | All-cause mortality | | | | Relapse | | | |
| Variable | Categories | N event/  total | HR (95% CI)^1^ | P | p_het_^2^ | N event/  total | HR (95% CI)^3^ | p | p_het_^2^ |
| Cytogenetics | Normal | 127/ 157 | 2.78 (1.48-5.21) | 0.002 |  | 86/157 | 3.75 (1.98-7.08) | <0.0001 |  |
| Age at HCT | Age≤40 | 123/ 158 | 2.31 (1.02-5.24) | 0.045 | 0.79 | 97/ 157 | 2.27 (1.03-5.01) | 0.04 | 0.82 |
|  | Age>40 | 330/ 378 | 2.63 (1.56-4.43) | 0.0003 |  | 236/ 378 | 2.54 (1.43-4.50) | 0.001 |  |
| 1. Models for all-cause mortality were adjusted for recipient race, Karnofsky Performance Status scores, study cohort, GvHD prophylaxis, donor-recipient CMV serostatus matching, and graft type. 2. P_het_=P_heterogeneity_ 3. Models for relapse were adjusted for Karnofsky Performance Status scores and graft type. | | | | | | | | | |

# **Supplemental Table 5.** Subgroup analysis of the association between CNLOH in chr13q or 17p and outcomes after HCT in patients with *De Novo* AML.

| Remission status | Copy neutral  loss-of heterozygosity | All-cause mortality | | | | Relapse | | | |
| --- | --- | --- | --- | --- | --- | --- | --- | --- | --- |
|  |  | Total | Event | HR (95% CI) | p | Total | Event | HR (95% CI) | p |
| Not in CR | chr13q | 455 | 377 | 2.82 (1.81-4.40)^1^ | <.0001 | 454 | 278 | 2.86 (1.78-4.62)^2^ | <.0001 |
| In CR | chr17p | 1156 | 719 | 3.14 (1.38-7.15)^3^ | 0.01 | 1156 | 411 | 2.33 (0.74-7.32)^4^ | 0.15 |

1. Model was adjusted for recipient race, Karnofsky Performance Status scores, study cohort, GvHD prophylaxis, donor-recipient CMV serostatus matching, and graft type.
2. Model was adjusted for Karnofsky Performance Status scores, and graft type.
3. Model was adjusted for recipient age, donor age, GvHD prophylaxis, Karnofsky Performance Status scores, donor-recipient CMV serostatus matching, and stratified on recipient sex, conditioning intensity, graft type, and year of transplant.
4. Model was adjusted for conditioning intensity, donor sex, study cohort, and stratified on Karnofsky Performance Status scores.

# **Supplemental Figure 1.** Chromosomal regions commonly affected by CNLOH in chr13q or 17p.

1. Chr17p CNLOH


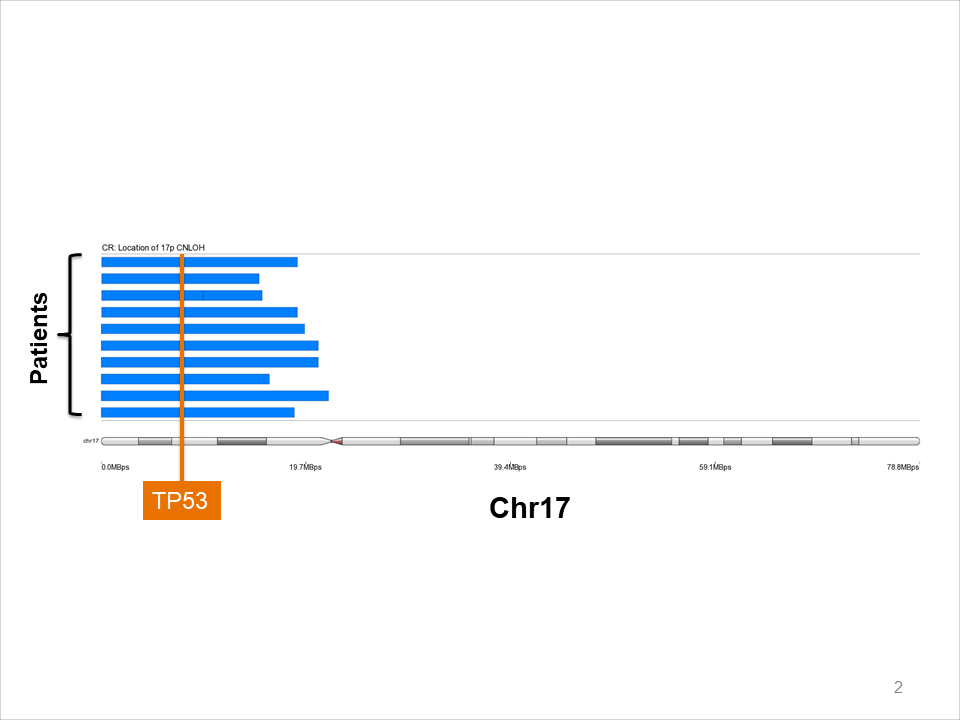


1. Chr13q CNLOH


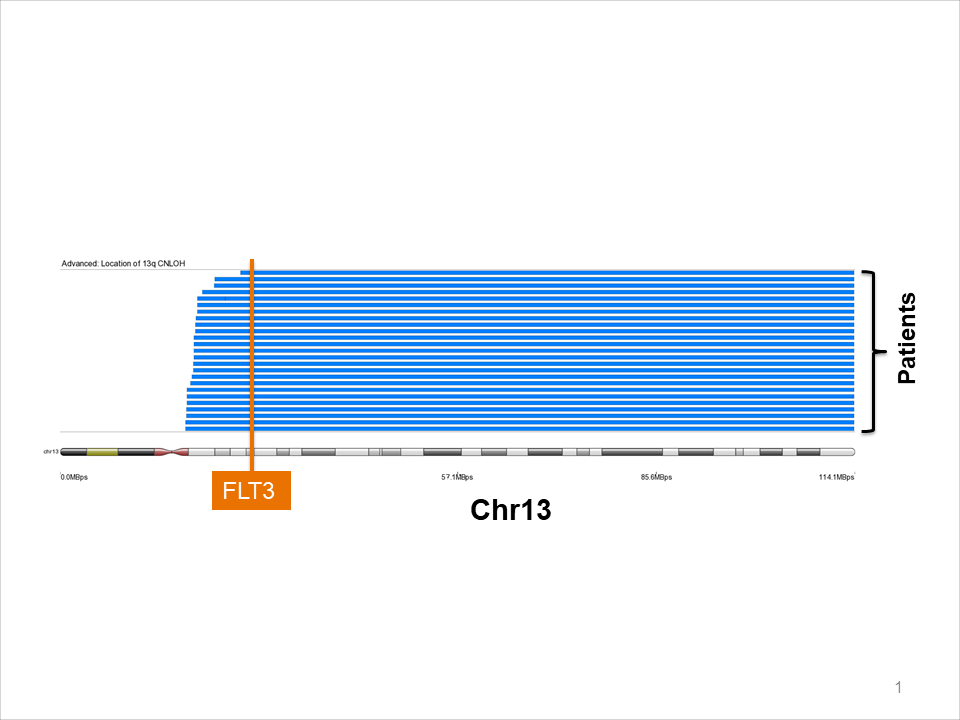


| **Supplemental Figure 2.** Cytogenetics at diagnosis reported by transplant centers in 43 patients with CNLOH in chromosome 13q or 17p |
| --- |
| 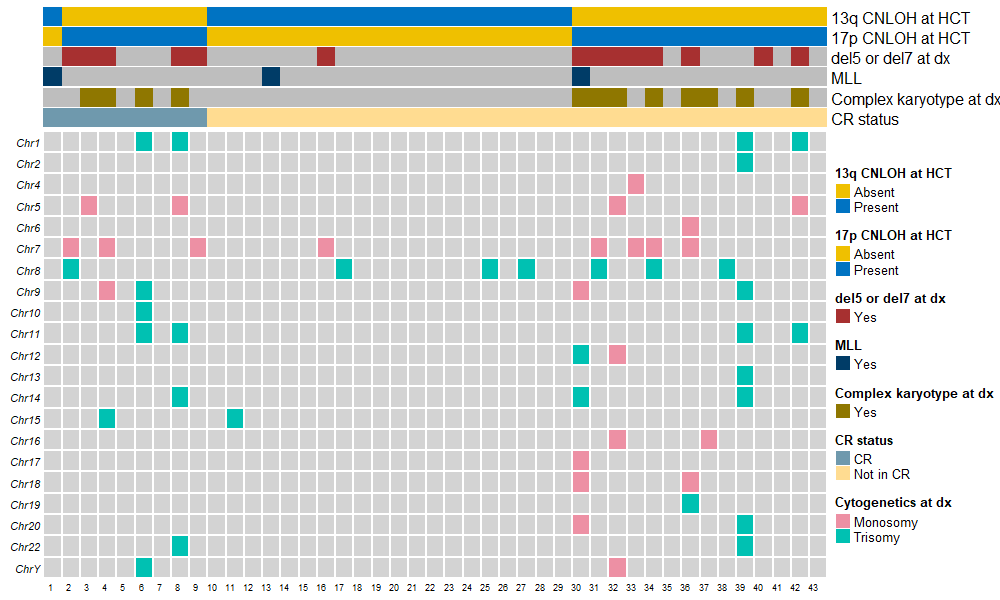 |
| CNLOH: Copy-neutral loss-of-heterozygosity, HCT: hematopoietic cell transplantation, del5: deletion of chromosome 5, del7: deletion of chromosome 7, dx: diagnosis, CR: complete remission |

| **Supplemental Figure 3.** Genomic location and type of chromosomal aberrations among AML patients with normal cytogenetics at diagnosis (A) not in remission and (B) in remission at transplant: Yellow: copy-gain, blue: copy-neutral loss of heterozygosity, red: copy-loss. R package “OmicCircos”, version 1.28.0^1^ was used to create the figures. | |
| --- | --- |
| **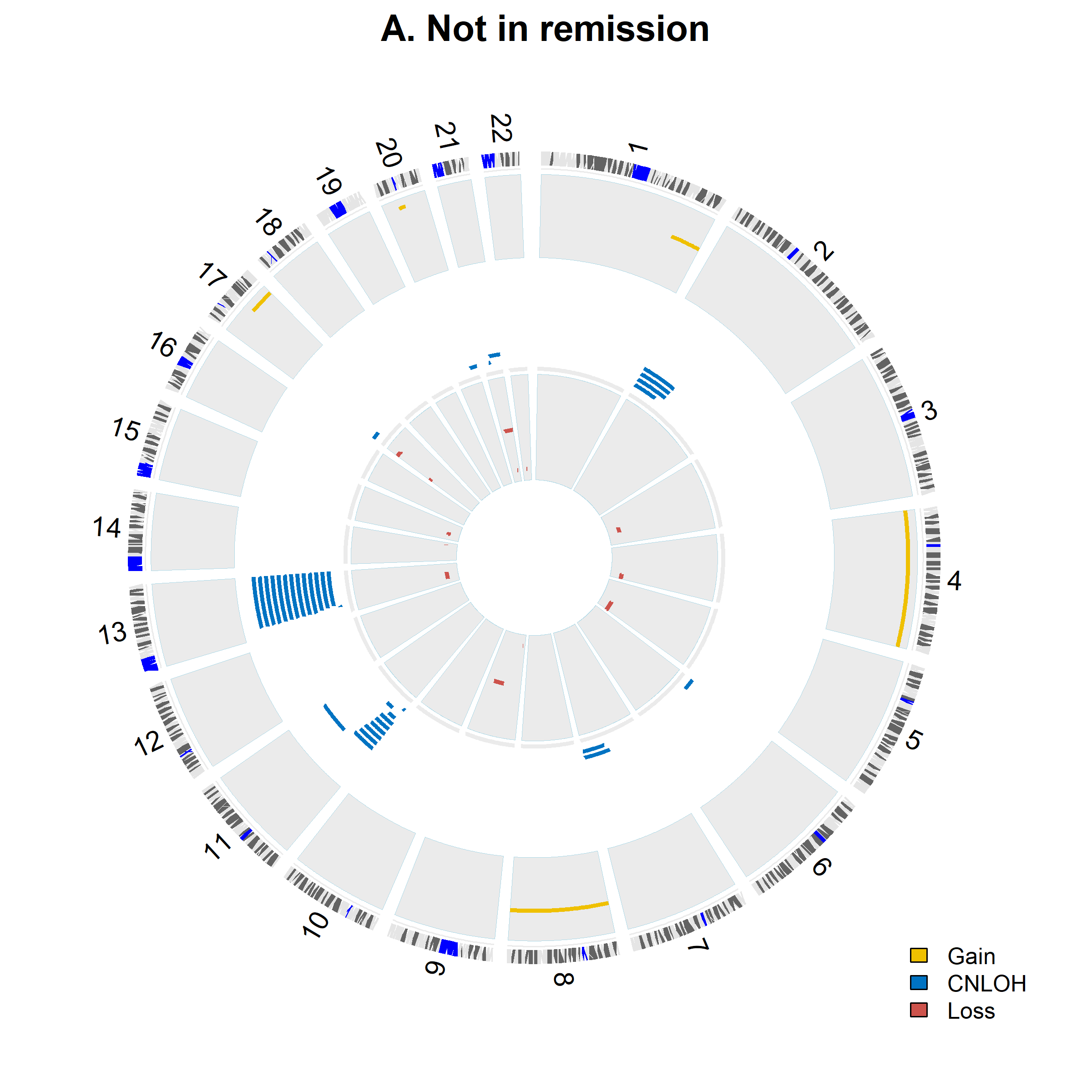** | **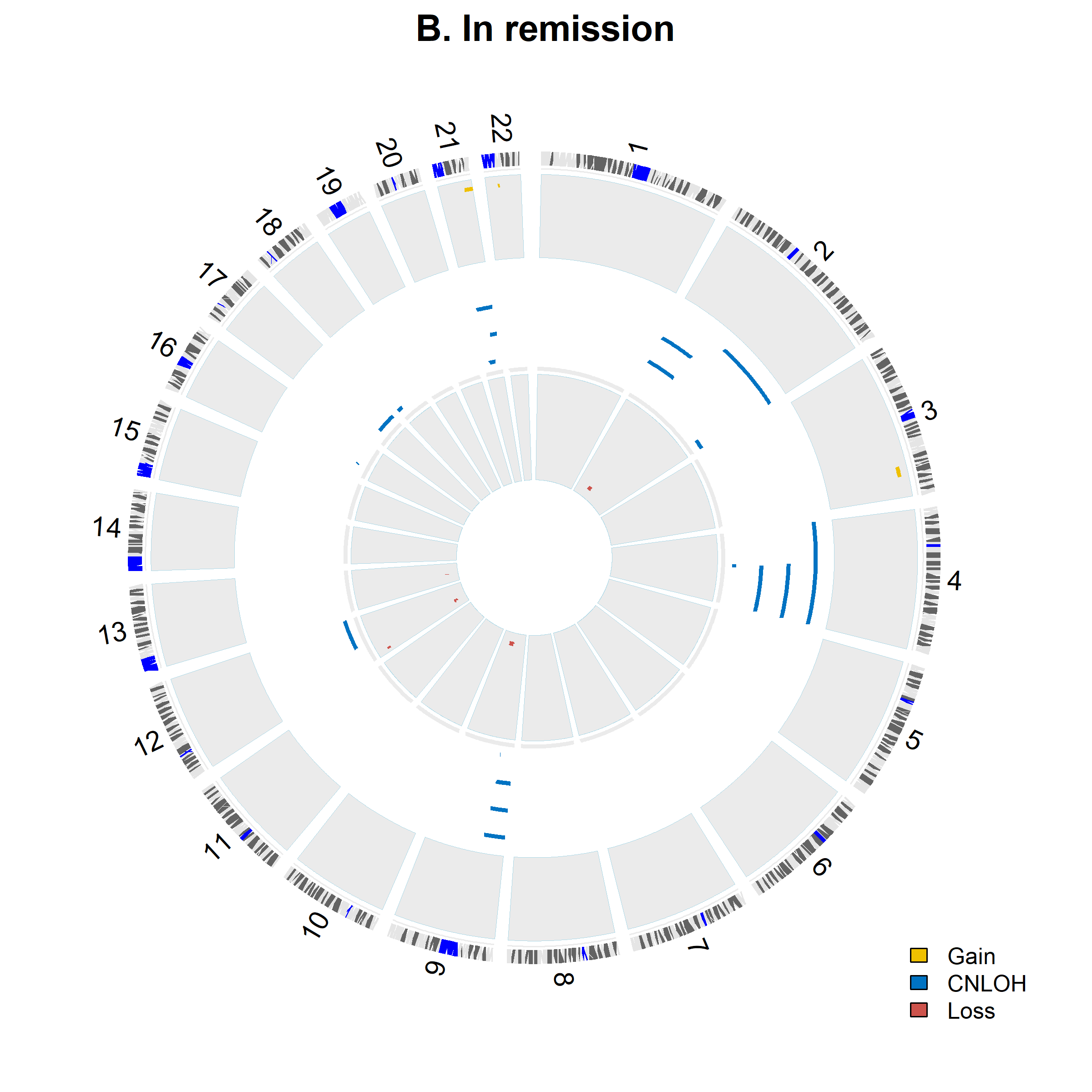** |

**Supplemental Figure 4.** Genomic location and type of chromosomal aberrations among AML patients by age at transplant. (A)-(C) not in remission and (D)-(F) in remission at transplant: Yellow: copy-gain, blue: copy-neutral loss of heterozygosity, red: copy-loss. R package “OmicCircos”, version 1.28.0^1^ was used to create the figures.

| **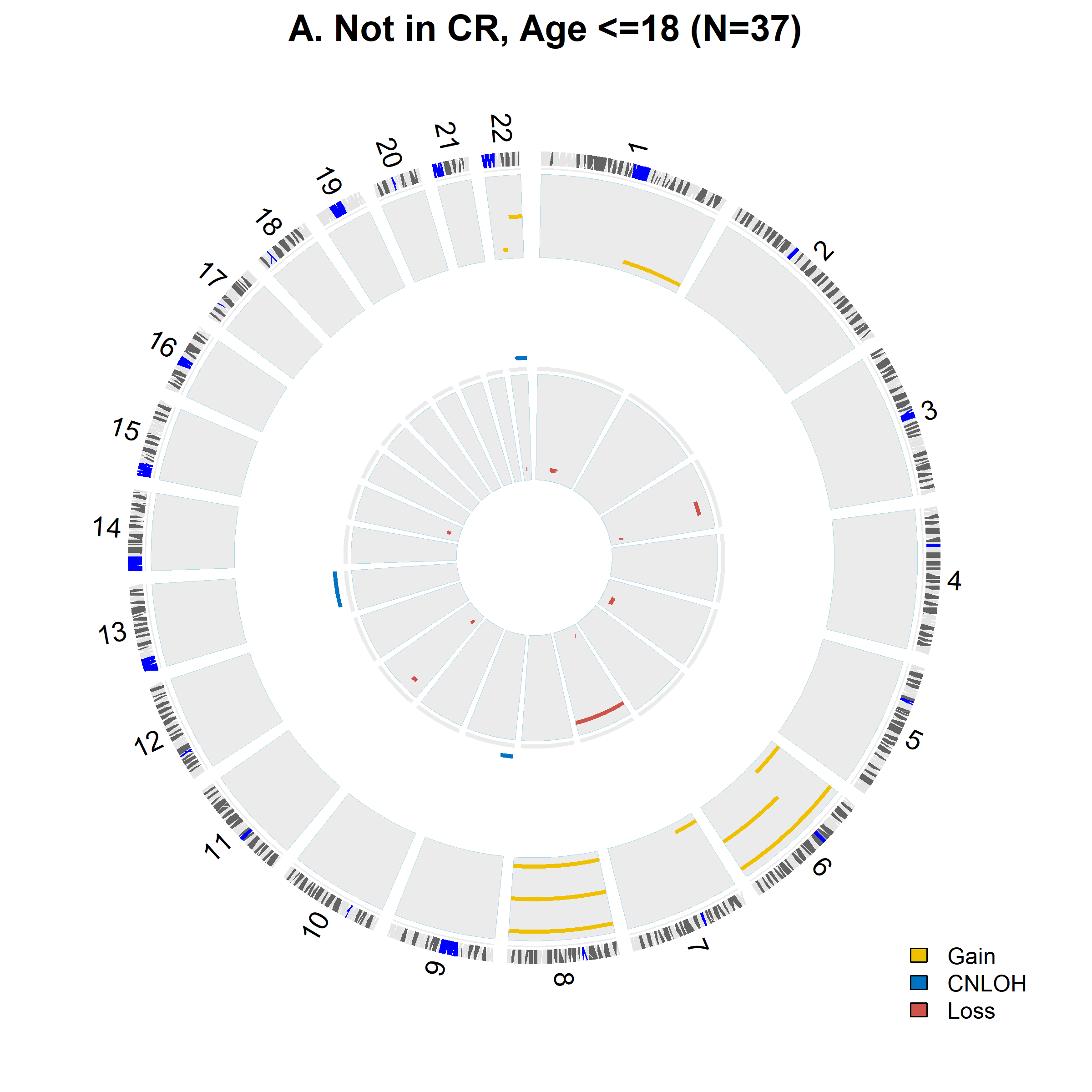** | **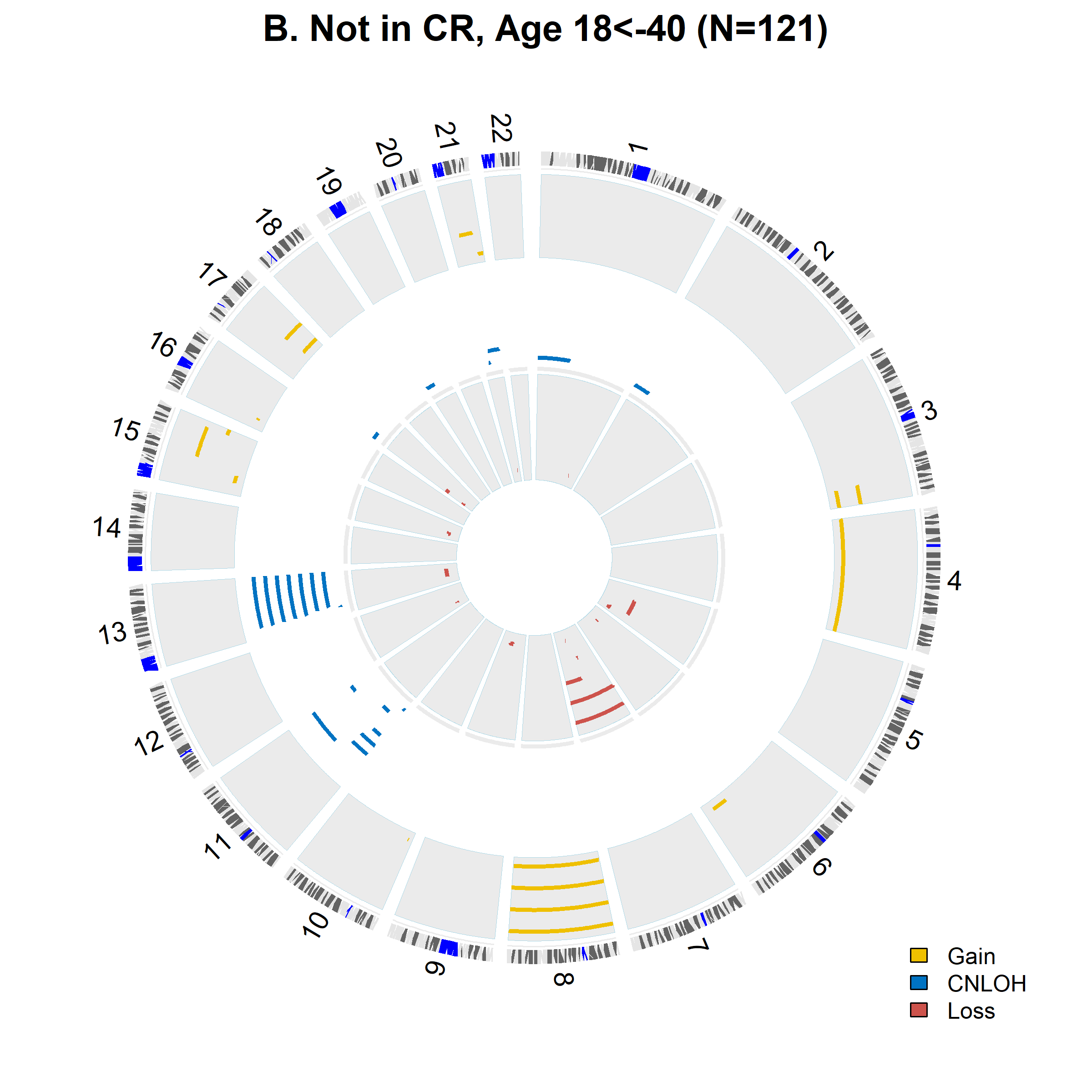** | **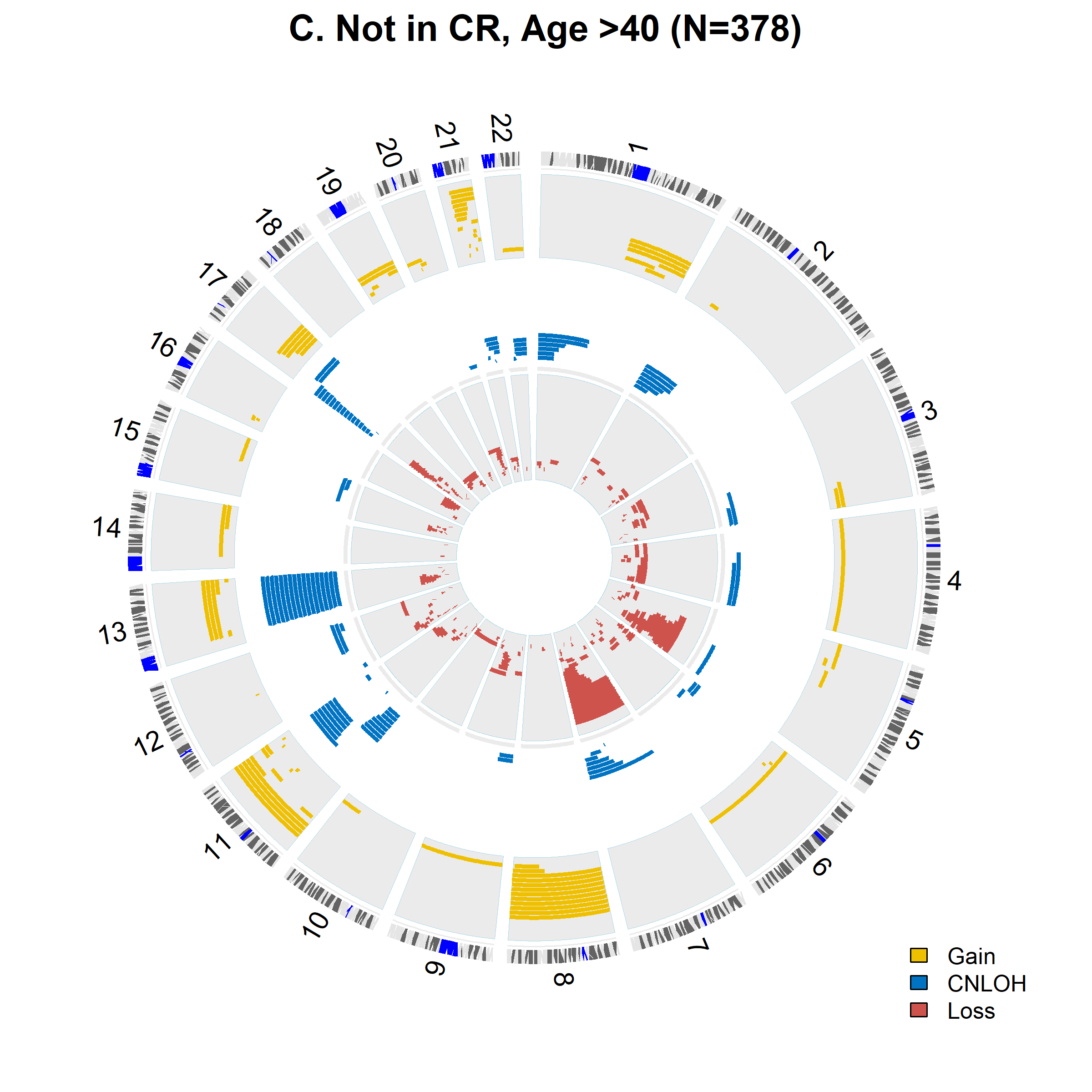** |
| --- | --- | --- |
| **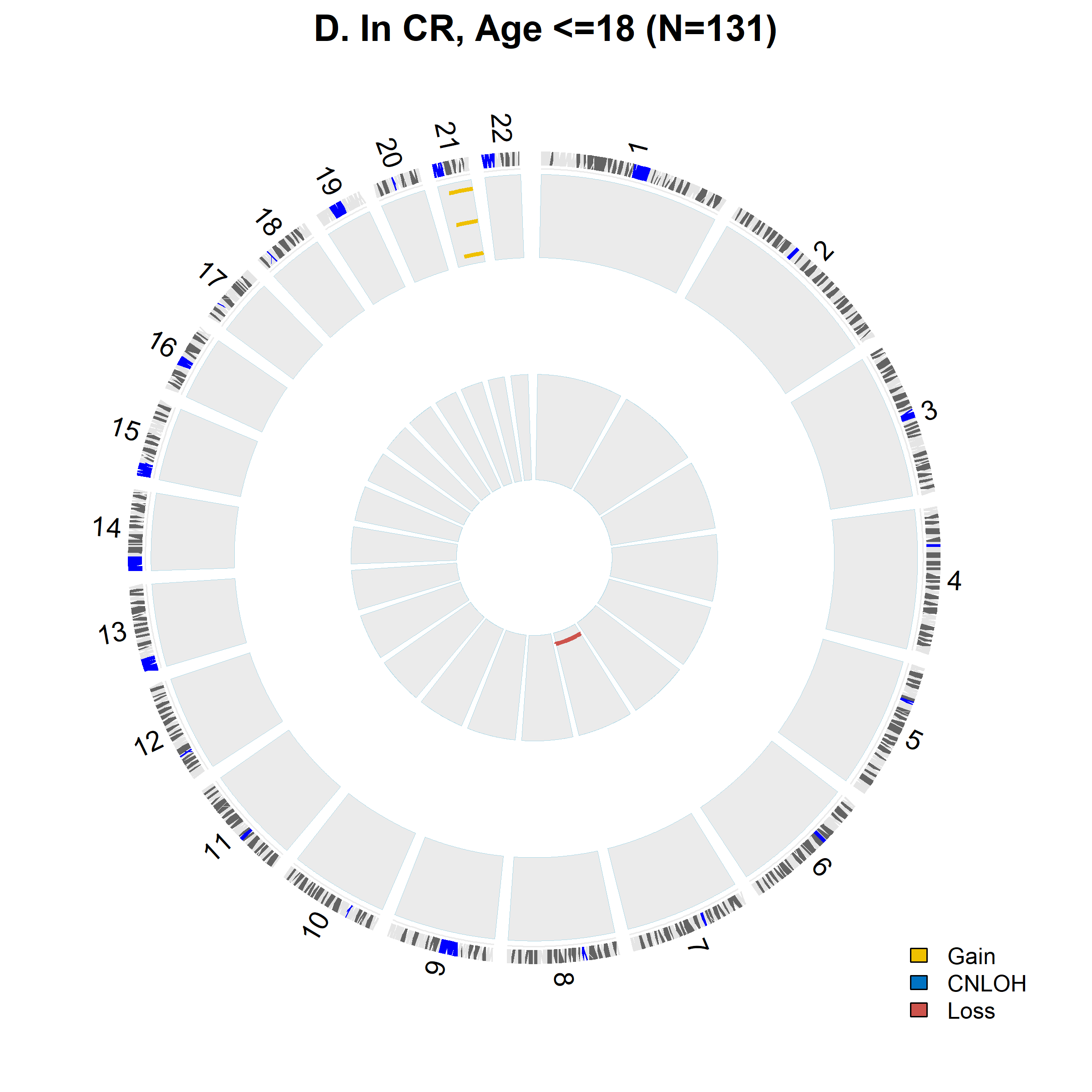** | **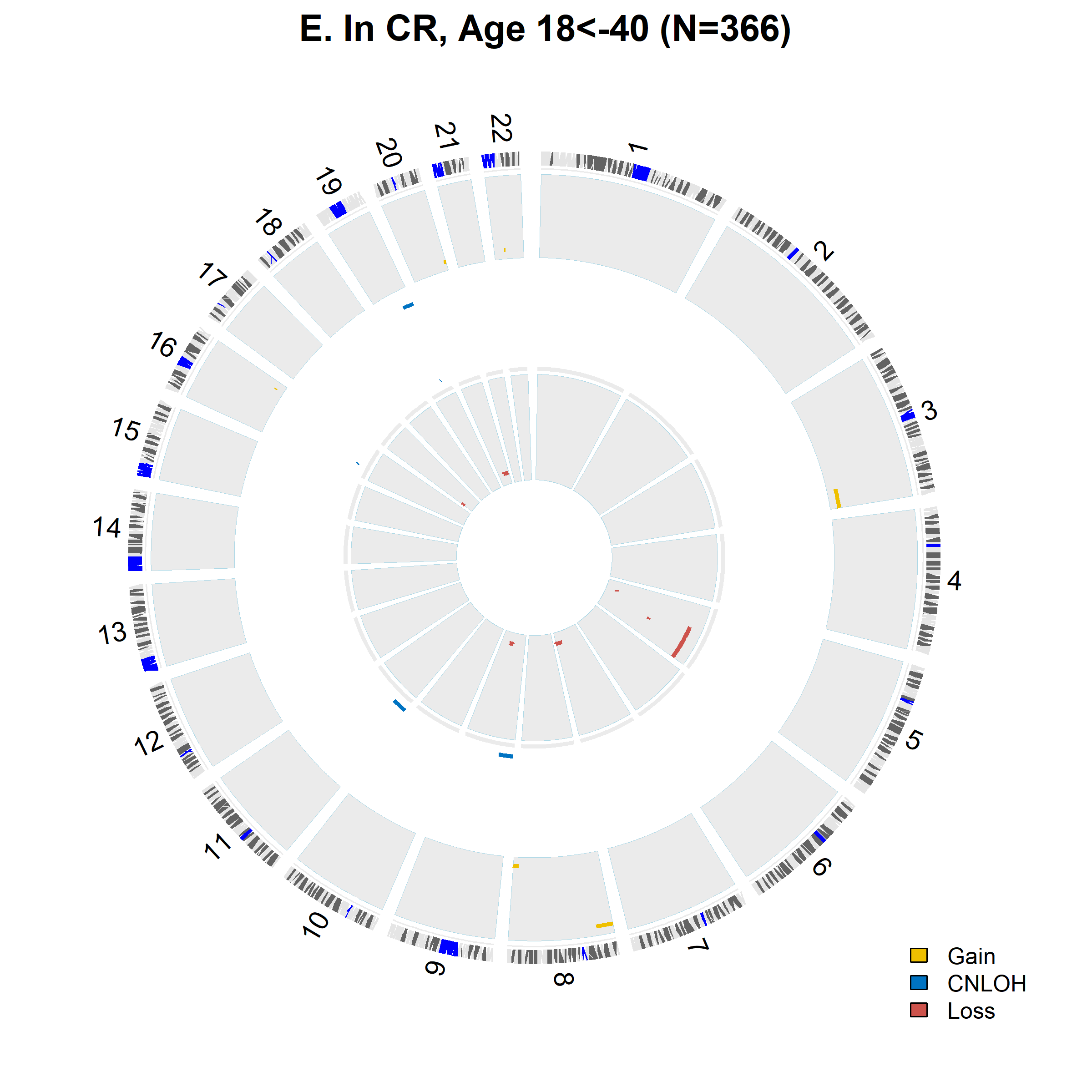** | **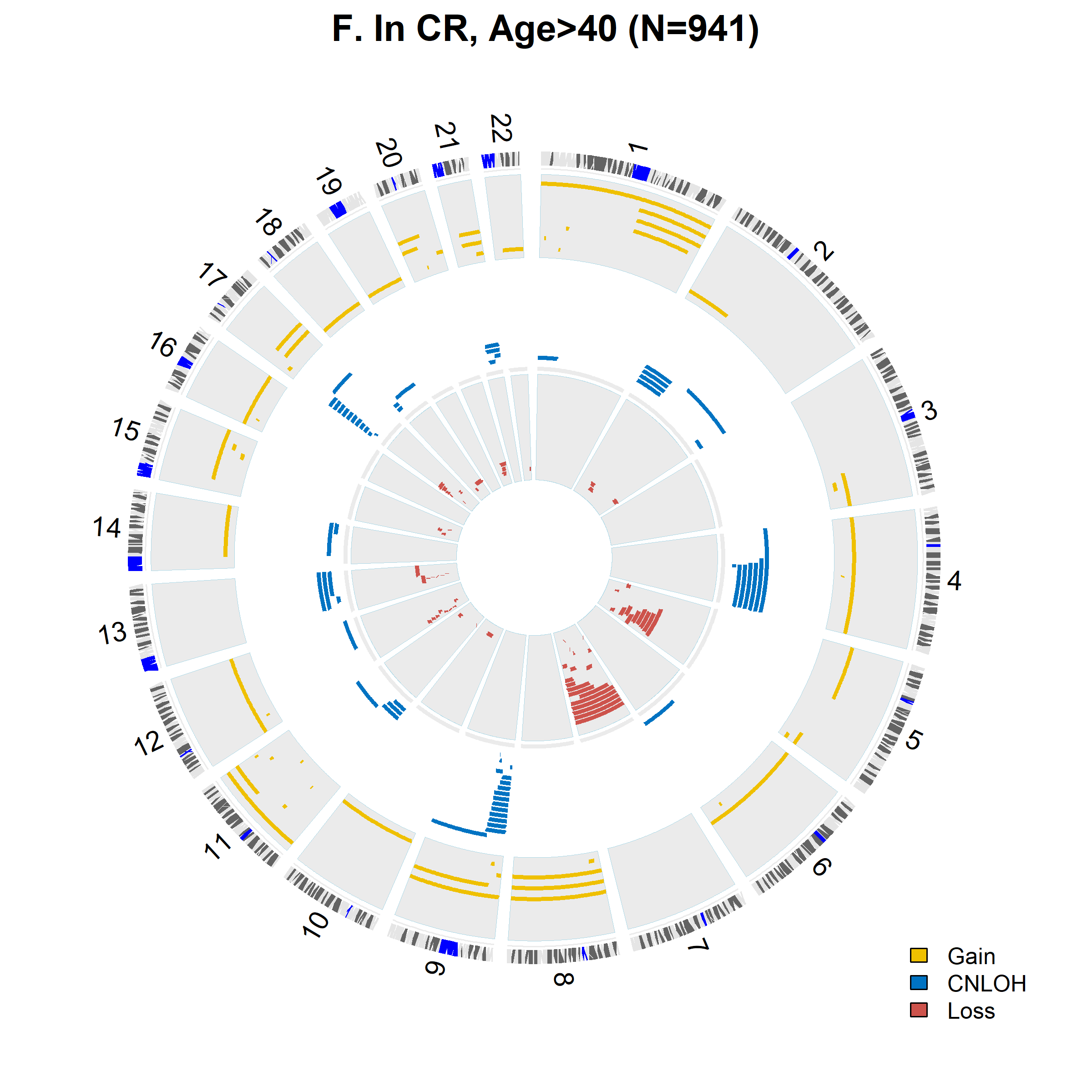** |

**Supplemental Figure 5.** Genomic location and type of chromosomal aberrations among AML patients by AML type. (A)-(B) not in remission and (C)-(D) in remission at transplant: Yellow: copy-gain, blue: copy-neutral loss of heterozygosity, red: copy-loss. R package “OmicCircos”, version 1.28.0^1^ was used to create the figures.

| A. Advanced, *De Novo* | B. Advanced, treatment-related |
| --- | --- |
| 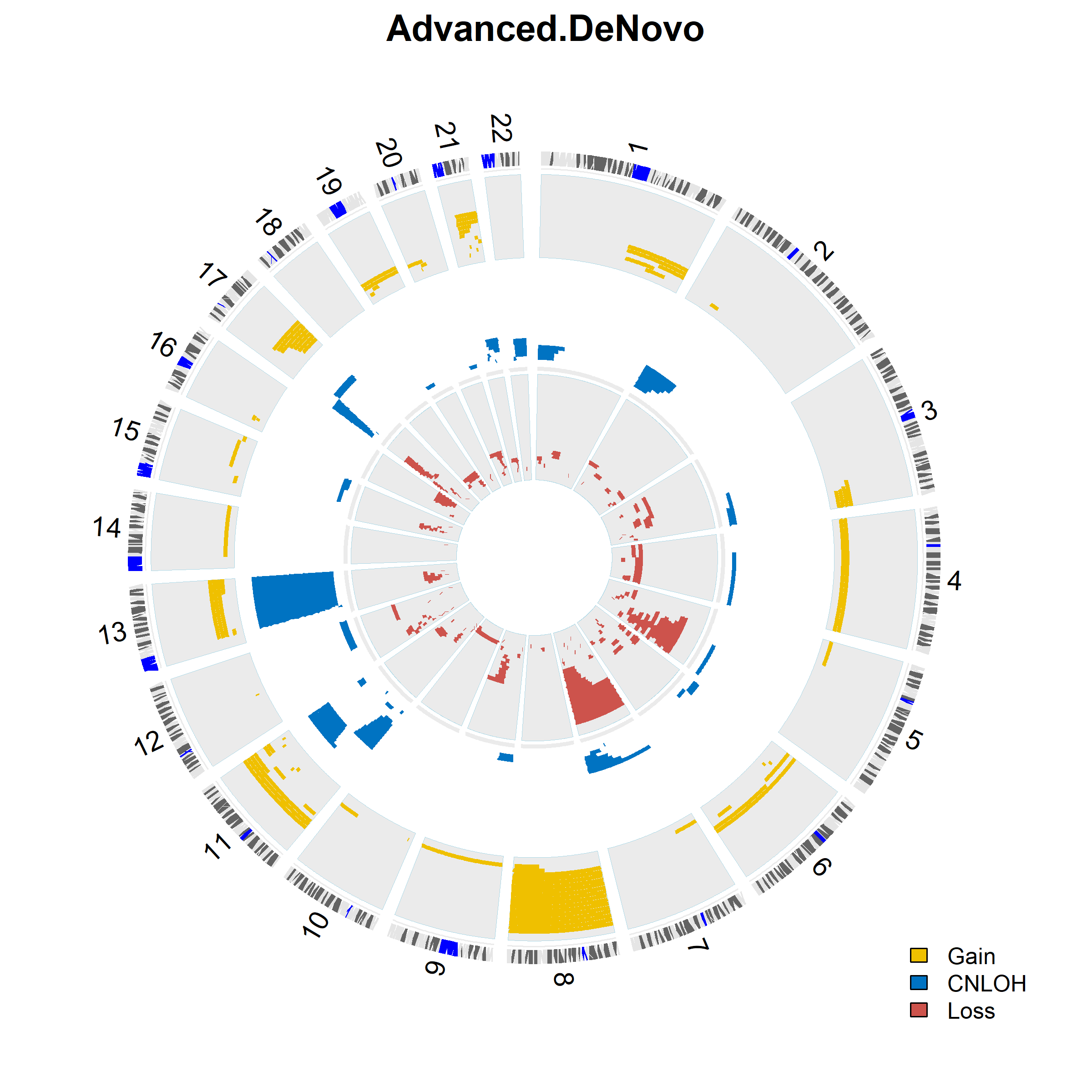 | 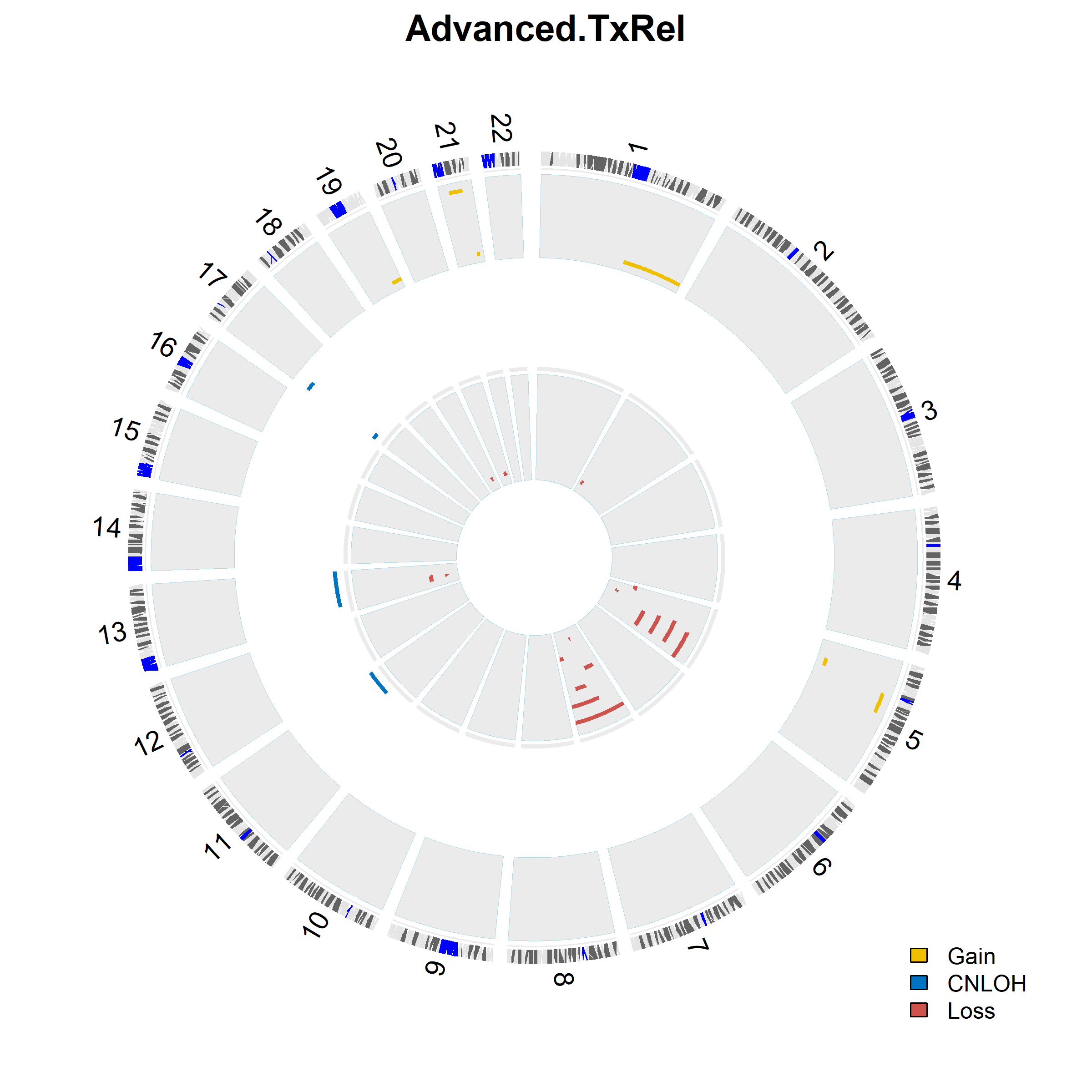 |
| C. In remission, *De Novo* | D. In remission, treatment-related |
| 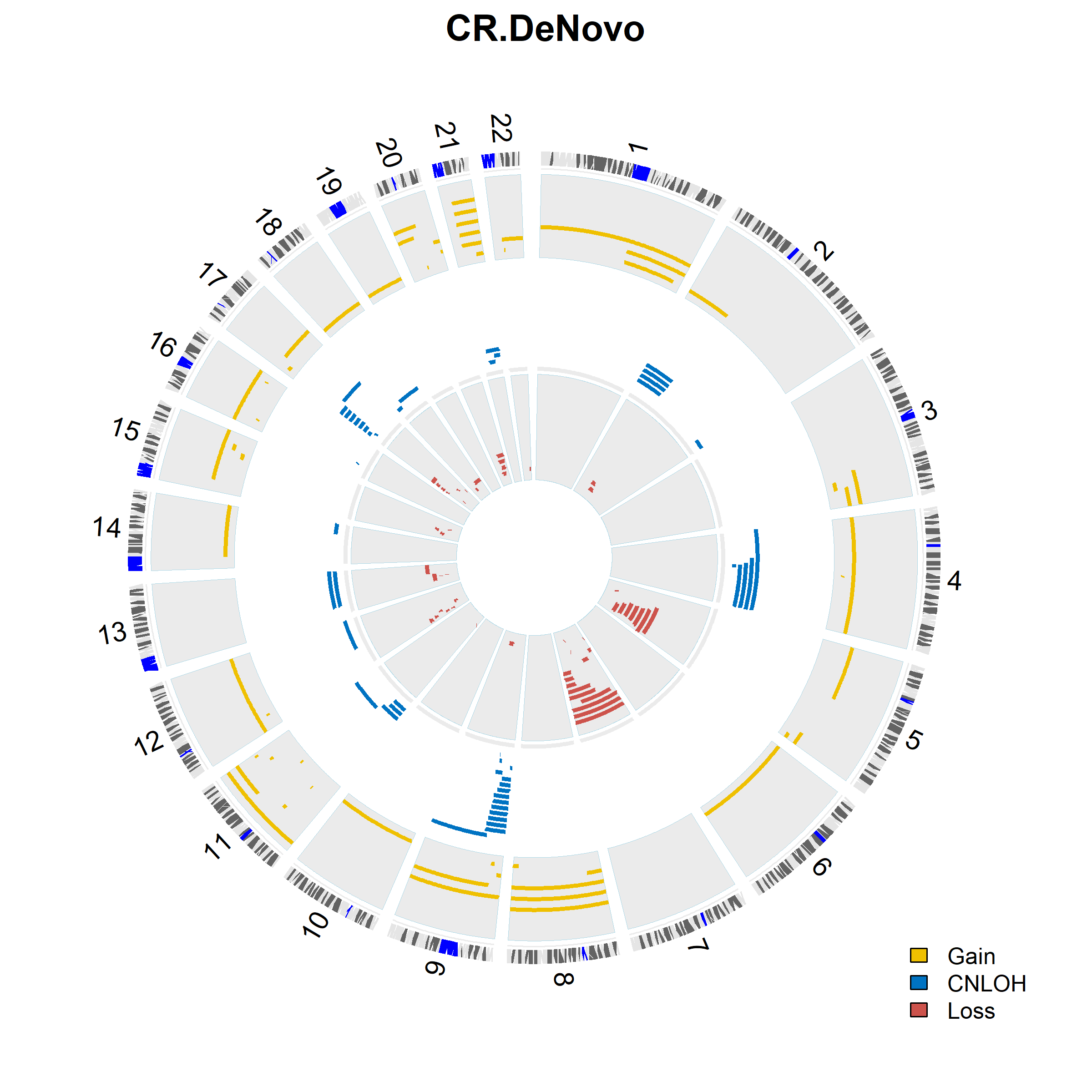 | 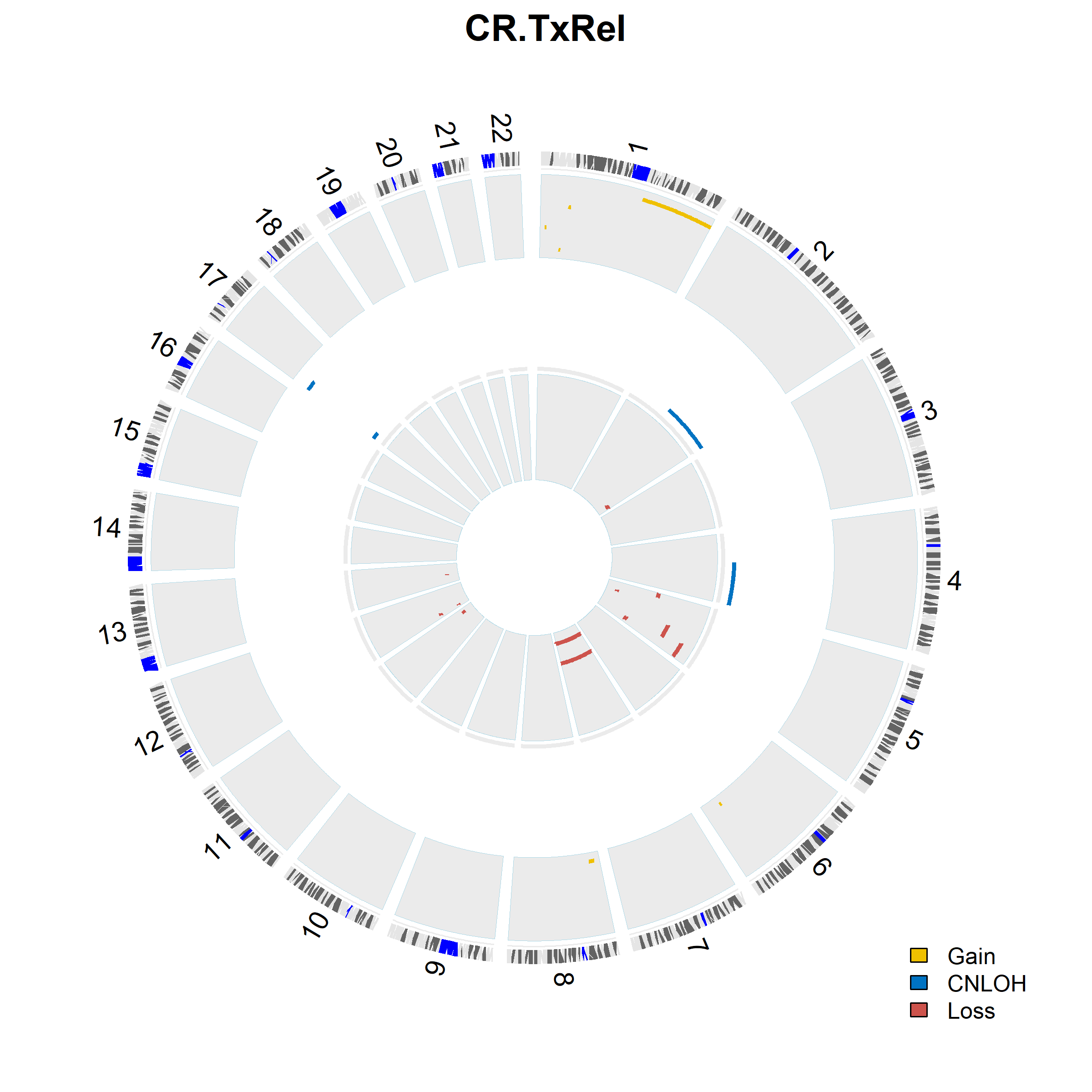 |

**Reference**

1. Hu Y, Yan C. (2020). OmicCircos: High-quality circular visualization of omics data. R package (Version 1.28.0) <https://bioconductor.org/packages/OmicCircos/>.
